# A NeuroD1 AAV‐Based Gene Therapy for Functional Brain Repair in Alzheimer's Disease‐Like Non‐Human Primate Model

**DOI:** 10.1002/advs.202520239

**Published:** 2026-03-10

**Authors:** Zhouquan Jiang, Yongpeng Qin, Bin Luo, Fan Bai, Jiangyue Liu, Long Ma, Shu He, Rongjie Chen, Yuchen Wang, Shanggong Liu, Ying Sun, Yi Chen, Shuo Zhang, Jiaqi Liang, Feng Liao, Huiyi Wei, Junjie Wei, Lu Wang, Hao Xu, Zheng Wu, Gong Chen, Wenliang Lei

**Affiliations:** ^1^ State Key Laboratory of Bioactive Molecules & Druggability Assessment Guangdong Basic Research Center of Excellence for Natural Bioactive Molecules & Discovery of Innovative Drugs Guangdong Key Laboratory of Non‐Human Primate Research State Key Laboratory of CNS Regeneration (Ministry of Education) GHM Institute of CNS Regeneration Jinan University Guangzhou Guangdong China; ^2^ Department of Nuclear Medicine and PET/CT‐MRI Centre the First Affiliated Hospital Jinan University Guangzhou Guangdong China

**Keywords:** Alzheimer's disease, hippocampal atrophy, NeuroD1, neuroregeneration, neuroinflammation, non‐human primate, spatial working memory

## Abstract

There is a pressing demand for neuroregenerative treatment for Alzheimer's disease (AD). Recently, a NeuroD1‐mediated neuroregeneration strategy has been proposed, yet its efficacy remains untested in non‐human primate (NHP) AD models closely reflecting human pathology. This study evaluates the therapeutic potential of NeuroD1 AAV‐based gene therapy in an NHP AD model with hippocampal hTau overexpression, utilizing immunostaining, fluorescence/confocal imaging, MRI and FDG PET scans, Simoa CSF biomarker analysis, behavioral tests, and bulk RNA sequencing. NeuroD1 AAV‐based gene therapy prevents neuronal damage and degeneration, inhibits hippocampal atrophy, and reduces neuroinflammation in NHP AD models. It also repairs vascular and BBB damage, restores CSF AD biomarker levels, improves hippocampal glucose metabolism, and enhances spatial working memory. Transcriptome analysis further reveals upregulated neuronal function and synaptic transmission, along with downregulated neuroinflammation and apoptosis. Collectively, our findings demonstrate that NeuroD1 AAV‐based gene therapy repairs and restores brain structure and function in NHP AD models, highlighting its therapeutic potential.

## Graphical abstract

This study tests NeuroD1 AAV‐based gene therapy in a non‐human primate Alzheimer's disease model. The therapy prevents neuronal damage, inhibits hippocampal atrophy, and reduces neuroinflammation. It also repairs vascular and blood‐brain barrier damage, restores cerebrospinal fluid biomarkers, enhances hippocampal glucose metabolism, and improves spatial memory. Transcriptome analysis reveals enhanced neuronal function and reduced neuroinflammation, supporting its therapeutic potential.

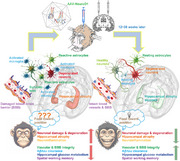

## 1 Introduction

Alzheimer's disease is the leading cause of dementia and the most prevalent neurodegenerative disorder, with its prevalence steadily rising as global populations age. This devastating disease poses far‐reaching social consequences, including diminished quality of life for patients, increased caregiver burden and stress, and substantial healthcare costs for families and societies [1].

Current treatments for AD mainly include pharmacological therapies and immunotherapies. The primary medications prescribed are cholinesterase inhibitors (Donepezil, Rivastigmine, and Galantamine) and Memantine (an NMDA receptor antagonist), which aim to improve cognitive function, memory, and behavior by modulating neurotransmitters [2, 3]. However, these drugs only provide modest symptomatic relief, do not halt disease progression, and their effectiveness diminishes over time. Additionally, they also come with side effects that can affect patients’ quality of life, and they may not work for all individuals, especially those in advanced stages [4]. Immunotherapies aim to harness the immune system to target pathological proteins like amyloid‐beta (Aβ) and tau, which are central to AD pathology. These include active immunotherapies with vaccines like AN1792 and CAD106, and passive immunotherapies with monoclonal antibodies (mAbs) like aducanumab, lecanemab, and donanemab [5, 6, 7]. While these immunotherapies have shown varying success in reducing Aβ burden and slowing cognitive decline, their clinical applications are limited by serious adverse effects (such as meningitis), mixed results in improving cognitive outcomes, and uncertain effects on disease progression [8, 9]. Overall, while current treatments offer temporary symptom relief for some patients, their limitations highlight the urgent need for more effective, disease‐modifying therapies that directly address the underlying pathogenic mechanisms of AD. Since neuronal damage and loss are the primary causes of brain dysfunction in AD, effective disease‐modifying therapies must ultimately have the capacity to regenerate functional neurons to replace the damaged and dead ones [10].

In recent years, in situ glia‐to‐neuron conversion techniques, which convert glial cells in the central nervous system into functional neurons, have opened new avenues for innovative treatments for neurodegenerative disorders and injuries. Numerous research teams have successfully achieved glia‐to‐neuron conversion through the utilization of various transcription factors (including Ngn2, NeuroD1, Ascl1, Sox2,…), combinations of these factors, or the repression of the RNA‐binding protein Ptbp1 [11, 12]. Among many outstanding works, our previous study reported that NeuroD1, a transcription factor involved in neuronal differentiation and development, can directly convert reactive glial cells into functional neurons in vivo after brain injury in AD mouse models [13]. Moreover, additional research into the therapeutic potential of NeuroD1 in various neurological conditions has shown that NeuroD1 AAV‐based gene therapy can help restore brain structure and function in rodent models of ischemic stroke, Huntington's disease, and temporal lobe epilepsy, as well as in a monkey model of ischemic stroke. It's worth noting that NeuroD1 AAV‐based gene therapy not only regenerated new neurons that integrated into existing brain circuits, but also reduced neuroinflammation, protected damaged neurons, improved motor performance, and alleviated cognitive impairments in these preclinical studies [14, 15, 16, 17].

In this study, we delivered AAVs encoding the neural transcription factor NeuroD1 into the hippocampus of NHPs exhibiting AD‐like pathologies [18]. We observed widespread expression of NeuroD1 throughout the hippocampus of these AD‐like monkeys. The NeuroD1 AAV‐based gene therapy not only prevented neuronal damage and loss, but also halted the progression of hippocampal atrophy. Additionally, this gene therapy alleviated neuroinflammation, restored vascular integrity, and normalized CSF AD biomarker levels. Furthermore, this gene therapy improved glucose metabolism and alleviated working memory deficits, as demonstrated by cognitive behavioral assessments. Mechanistically, transcriptome analysis revealed that NeuroD1 AAV‐based gene therapy upregulated pathways involved in neuronal and synaptic functions, while downregulating those associated with neuroinflammation and apoptosis. Collectively, these findings suggest that NeuroD1 AAV‐based gene therapy holds promise as an effective therapeutic strategy for the treatment of AD.

It is worth noting that although glia‐to‐neuron conversion via transcription factors like NeuroD1 holds promise for treating neurodegenerative diseases and CNS injuries, debates continue over its specificity, efficiency, and reliability, with some attributing the newly observed neurons to pre‐existing ones. Consensus remains elusive on many fundamental aspects, including optimal reprogramming conditions, conversion rates, molecular mechanisms, lineage‐tracing methods, and neuronal maturity and integration [19, 20, 21, 22, 23, 24]. A most recent study by Richard Lu's group demonstrated that induced‐expression of NeuroD1 in lineage‐traced astrocytes clearly converted astrocytes into neurons, upholding the capability of NeuroD1 conversion [25]. In this context, this NeuroD1 AAV‐based gene therapy study in an AD‐like NHP model fully acknowledges the multifactorial and complex nature of its therapeutic outcomes. Specifically, the observed benefits likely result from the combined effects of multiple mechanisms, including neuroregeneration, neuroprotection, inflammation reduction, BBB repair, toxic protein clearance, and microenvironment modulation. The relative contribution and intricate interplay of these mechanisms remain to be fully elucidated. Worldwide efforts, including our team's, are actively employing multi‐omics bioinformatics, lineage‐tracing models, and in vivo imaging to provide deeper, more definitive insights for glia‐to‐neuron conversion. We anticipate that these concerted efforts will advance in situ glial reprogramming technologies and treatments of neurodegenerative diseases and CNS injuries.

## 2 Results

### 2.1 Establishment of an NHP Model with AD‐Like Pathology Through Hippocampal Overexpression of hTau

Rodent AD models, while showing many successes, bear significant limitations such as much shorter lifespan, cognitive testing difficulty, different brain structure and tau pathology from AD patients [26, 27]. NHPs, on the other hand, share genetic, anatomical, physiological and pathological similarities with humans, and hence have emerged as important tools for investigating AD pathogenesis and therapeutic interventions [28, 29]. In recent decades, NHP models for AD, particularly transgenic primates, have made significant progress, offering valuable insights into AD pathology and therapeutic testing. However, challenges such as high costs, ethical concerns, long lifespans, and limited translational relevance hinder their widespread use [30].

**FIGURE 1:**
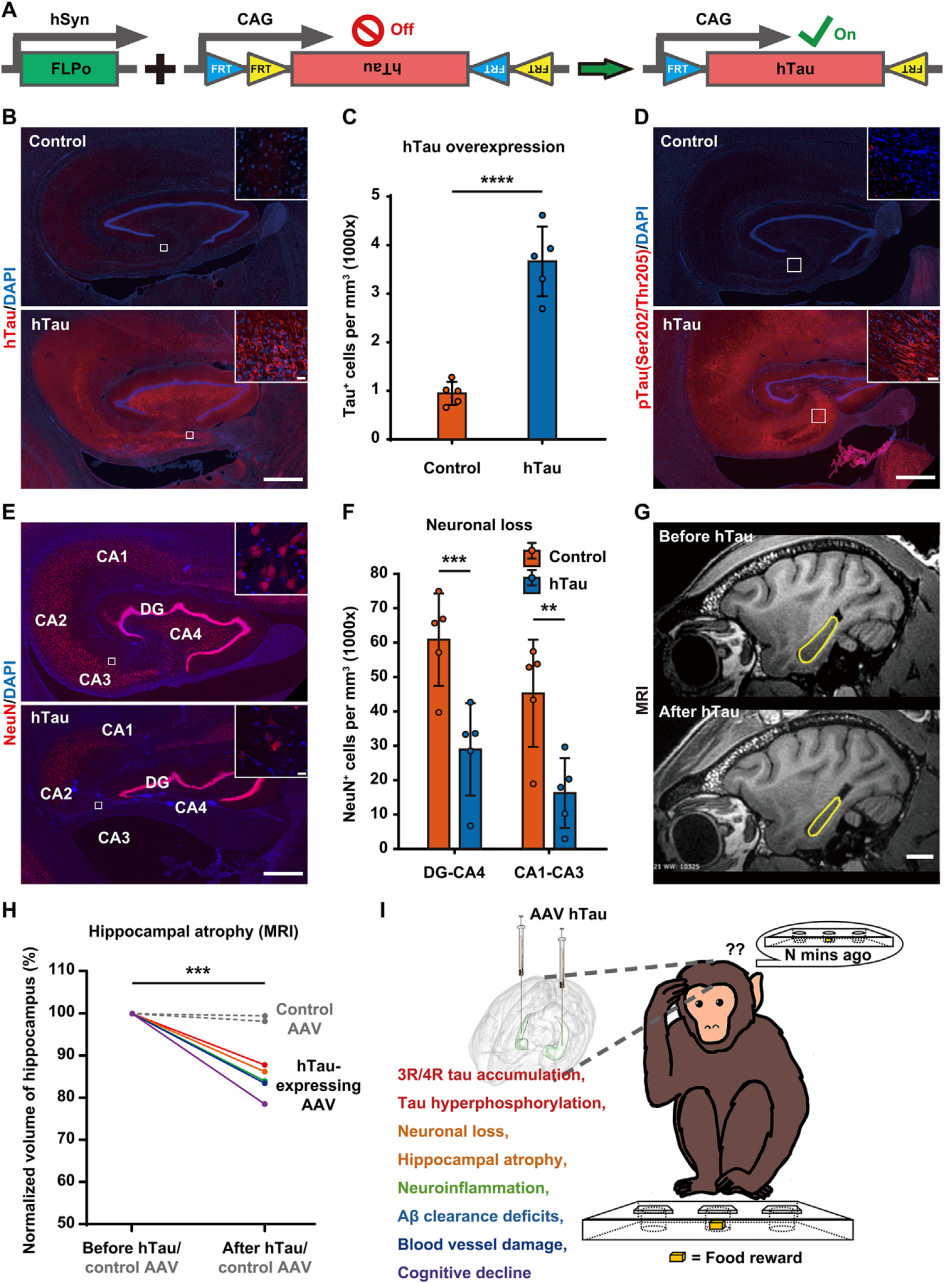
Development of an NHP model with AD‐like pathology. A) A schematic diagram depicts the engineered AAVs (Syn::FLPo and CAG::FRT‐hTau) that have been designed to express hTau upon activation by the FLPo recombinase. B) Representative images of tau immunostaining demonstrate elevated tau expression in the hippocampus of AD‐like monkeys compared to normal monkeys following AAV‐induced tau overexpression. Scale bars, 1 mm and 20 µm (inset). C) Quantitation of the density of Tau^+^ monkey hippocampal cells indicates a significantly higher hTau expression level in AD‐like monkeys versus normal monkeys. ****p < 0.0001, Student's *t* test, *N* = 5. D) Representative images of phospho‐tau immunostaining reveal the accumulation of phosphorylated tau (Ser202/Thr205) in the hippocampus of AD‐like monkeys following hTau overexpression. Scale bars, 1 mm and 50 µm (inset). E) Representative images of NeuN immunostaining demonstrate notable neuronal loss and hippocampal atrophy following hTau overexpression. Scale bar, 1 mm and 20 µm (inset). F) Quantitation of NeuN positive cells in the hippocampus indicates a significant decrease in their number in both the DG‐CA4 and CA1‐CA3 regions after hTau overexpression. ***p < 0.001, **p < 0.01, Student's *t* test, *N* = 5. G) Representative sagittal planes of T1‐weighted MRI images reveal a reduction in hippocampal volume after hTau overexpression in the same monkey, with the hippocampal region outlined by yellow lines. Scale bar, 1 cm. H) Quantitation of the normalized hippocampal volume indicates a significant shrinkage of the hippocampus following hTau overexpression, with each color representing a distinct animal. The gray dashed lines represent control animals injected with control AAVs. ***p < 0.001, Student's *t* test, AD‐like monkeys *N* = 5; control monkeys *N* = 2. I) A schematic diagram summarizes and illustrates the diverse AD‐like pathological phenotypes observed in our NHP AD models overexpressing hTau.

To evaluate the safety and efficacy of our NeuroD1 AAV‐based gene therapy, we generated an NHP model with AD‐like pathology by overexpressing human tau (hTau) in the bilateral hippocampi of adult rhesus monkeys as previously described [18]. Briefly, we injected a pair of AAV vectors (AAV Syn::FLPo + AAV CAG::FRT‐hTau) into 6 injection sites predetermined by the T1‐weighted MRI scans in the bilateral hippocampi of adult rhesus monkeys (7–15 years old) under monitored anesthesia. AAV Syn::FLPo will express site‐specific recombinase FLPo in neurons under the synapsin promoter, which will induce robust hTau expression from AAV CAG::FRT‐hTau under the strong ubiquitous CAG promoter via FRT recombination (Figure 1A). Around 10 weeks after AAV injection, we found that hippocampal tau expression level in the monkeys injected with hTau expressing AAVs was significantly higher than that in the control monkeys (Figure 1B). High power images revealed excessive tau accumulation in neuronal soma and apical dendrites of pyramidal neurons (Figure 1B, insets). Quantitatively, the density of Tau^+^ hippocampal cells in AD‐like monkeys was ∼4 times higher than that in control monkeys, measured 10 weeks after viral injection (Figure 1C). In brains affected by AD, hyperphosphorylation of tau at specific sites, such as S202 or T205 within a proline‐rich region, leads to neurofibrillary tangle (NFT) formation [31, 32]. Consistently, using phospho‐tau antibody AT8, we found that many hippocampal neurons showed robust phospho‐tau (S202/T205) staining in their somata and dendrites at 10 weeks after hTau overexpression (Figure 1D). Moreover, high‐magnification confocal imaging of Thioflavine S‐stained hippocampal tissue revealed NFT‐like aggregates within neurons of our AD‐like monkeys. These observations indicate that hippocampal pathology in this NHP model displays characteristics resembling the NFTs observed in AD patients (Figure S1, Supporting Information).

Progressive neurodegeneration is a common pathological feature of AD and highly correlates with cognitive decline in AD patients [33, 34]. After hTau overexpression, the NeuN immunoreactivity was substantially lower across the monkey hippocampus, compared with the control group without hTau expression (Figure 1E). High power images highlighted abnormal NeuN signals in the hippocampus of our AD‐like NHP model (Figure 1E, insets). Quantitatively, the number of NeuN^+^ cells in the hippocampal DG/CA4 and CA1/CA2/CA3 regions decreased by approximately 55% and 65% after hTau overexpression, suggesting significant hippocampal neuronal loss in this NHP AD model (Figure 1F and Figure S2, Supporting Information). Hippocampal atrophy often leads to profound amnesia, a key characteristic of AD, and is also strongly associated with the progression of mild cognitive impairment (MCI) to AD [35, 36]. Significant shrinkage of the hippocampus was observed after hTau overexpression in NHPs (Figure 1G, yellow outlines). Quantitatively, the hippocampal volume of AD‐like monkeys measured from the MRI scans exhibited a 12–23% decline ∼9 weeks after hTau overexpression, while the hippocampal volume of control monkeys remained unchanged, indicating hippocampal atrophy in this NHP AD model (Figure 1H). It is noteworthy that the observed AD‐like pathology is highly improbable to be attributed to the mechanical injury associated with stereotactic injections or to the overexpression of GFP. This conclusion is supported by the absence of detectable AD‐like pathology in some control monkeys, which underwent identical stereotactic injection procedures and expressed comparable, if not higher, levels of GFP from AAV‐driven expression [18]. Besides pathological features like tau hyperphosphorylation, neuronal loss and hippocampal atrophy, the AD‐like NHP model also displayed many other pathological characteristics of AD. These included 3R/4R tau accumulation, tau propagation, neuroinflammation, deficiency in Aβ clearance, vascular abnormalities and cognitive impairment (Figure 1I) [18], suggesting that we have developed a potentially suitable NHP model to further evaluate therapeutic interventions for AD.

### 2.2 Broad Expression of Neural Transcription Factor NeuroD1 Throughout the AAV‐Injected Monkey Hippocampus

**FIGURE 2:**
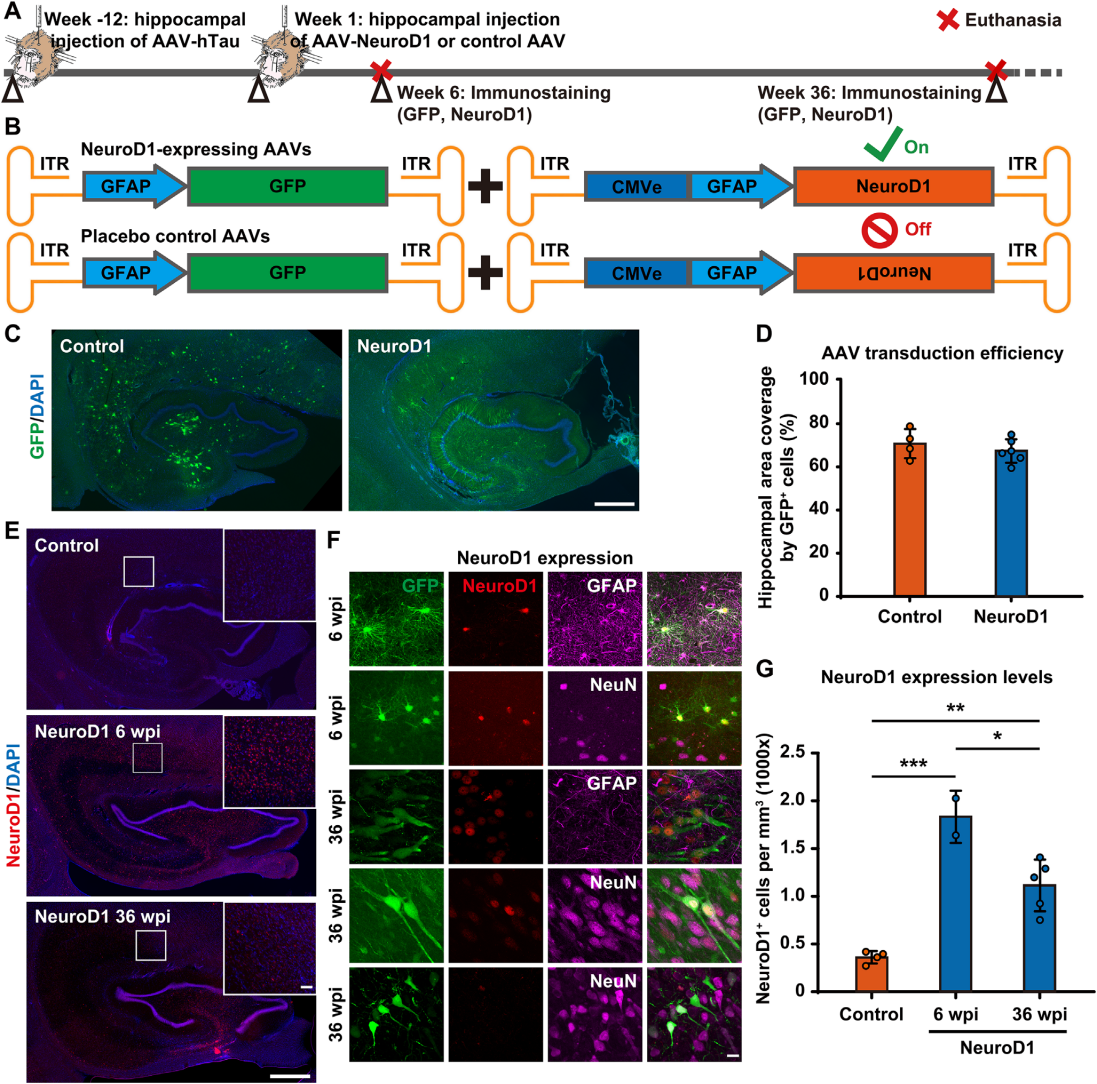
NeuroD1 overexpression throughout the monkey hippocampus after AAV‐based gene therapy. A) A schematic diagram illustrates the process of creating the NHP AD model, administering NeuroD1 AAV‐based gene therapy, and subsequently detecting the expression levels of the transcription factor NeuroD1. B) A schematic diagram depicts the engineered AAVs (GFAP(CMVe)::NeuroD1, GFAP(CMVe)::inverted NeuroD1, and GFAP::GFP) employed in both the gene therapy control group and NeuroD1 treatment group. C) Representative images of GFP immunostaining reveal that AAVs can effectively transduce most regions of the hippocampus. Please note that most GFP^+^ cells in the control group are astrocytes, while many GFP^+^ cells in the NeuroD1 treatment group are neurons. Scale bar, 1 mm. D) Quantitation of the hippocampal area coverage by GFP^+^ cells indicates that the genes delivered by the AAVs (GFP and NeuroD1) are expressed effectively throughout the monkey hippocampus. Control group *N* = 4, NeuroD1 group *N* = 6. E) Representative images of NeuroD1 immunostaining demonstrate effective NeuroD1 expression throughout the monkey hippocampus at both 6 and 36 weeks post AAV injection, with a notable reduction in NeuroD1 expression observed over time from 6 to 36 weeks following injection. Scale bars, 1 mm and 100 µm (inset). F) Representative images of NeuroD1/GFAP/NeuN immunostaining reveal that NeuroD1 is specifically expressed in astrocytes at 6 weeks post‐injection, while it is expressed in GFP^+^ neurons at 36 weeks post‐injection. Scale bar, 20 µm. G) Quantitation of the density of NeuroD1^+^ monkey hippocampal cells indicates high NeuroD1 expression in the monkey hippocampus at 6 weeks post‐injection, with a decline at 36 weeks post‐injection. *p < 0.05, **p < 0.01, ***p < 0.001, One‐way ANOVA with Tukey's post hoc test, Control group *N* = 4, NeuroD1 6 wpi group *N* = 2, NeuroD1 36 wpi group *N* = 5.

To investigate the efficacy of NeuroD1 AAV‐based gene therapy in our NHP AD model, we injected AAV9 vectors encoding NeuroD1 and GFP (GFAP(CMVe)::NeuroD1 + GFAP::GFP) or its non‐functional control version with inverted sequence and GFP (GFAP(CMVe)::inverted NeuroD1 + GFAP::GFP) in the monkey hippocampus at 12 weeks after hTau overexpression (Figure 2A,B; Table S1, Supporting Information). The AAV9 GFAP::GFP was employed to fluorescently label the astrocytes with GFP under the astrocytic promoter GFAP in the monkey hippocampus. The utilization of a strong CMV enhancer (CMVe) served to elicit a sufficiently high expression level of NeuroD1, which is crucial for astrocyte conversion [37, 38].

AAVs were injected at six evenly distributed injection sites determined by the T1‐weighted MRI scans. Thirty‐six weeks later, the GFP expression was still broadly detected across the hippocampus (Figure 2C), with ∼70% of hippocampal area covered by GFP^+^ cells (Figure 2D). NeuroD1 immunostaining clearly demonstrated hippocampal expression at both 6 and 36 weeks after AAV injection, with a notable decrease in overall expression level from 6 to 36 weeks post injection (Figure 2E, quantified in Figure 2G). Higher magnification images revealed that NeuroD1 expression was primarily detected in astrocytic nuclei at the 6‐week mark, whereas at 36 weeks, NeuroD1 expression was predominantly observed in neuronal nuclei (Figure 2F), suggesting potential conversion of NeuroD1‐expressing hippocampal astrocytes into neurons. Additionally, we observed GFP^+^ NeuN^+^ NeuroD1^−^ cells at 36 weeks, indicating downregulation of NeuroD1 by the GFAP promoter (Figure 2F). The decrease of NeuroD1 expression over time from 6 weeks to 36 weeks post injection is likely attributable to the downregulation of the astrocyte‐specific GFAP promoter activity following the potential astrocyte‐to‐neuron conversion. Collectively, these results demonstrated that the AAV‐based gene delivery method employed in this gene therapy can effectively achieve widespread expression of NeuroD1 throughout the monkey hippocampus.

### 2.3 Preventing Neuronal Degeneration by NeuroD1 AAV‐Based Gene Therapy

**FIGURE 3:**
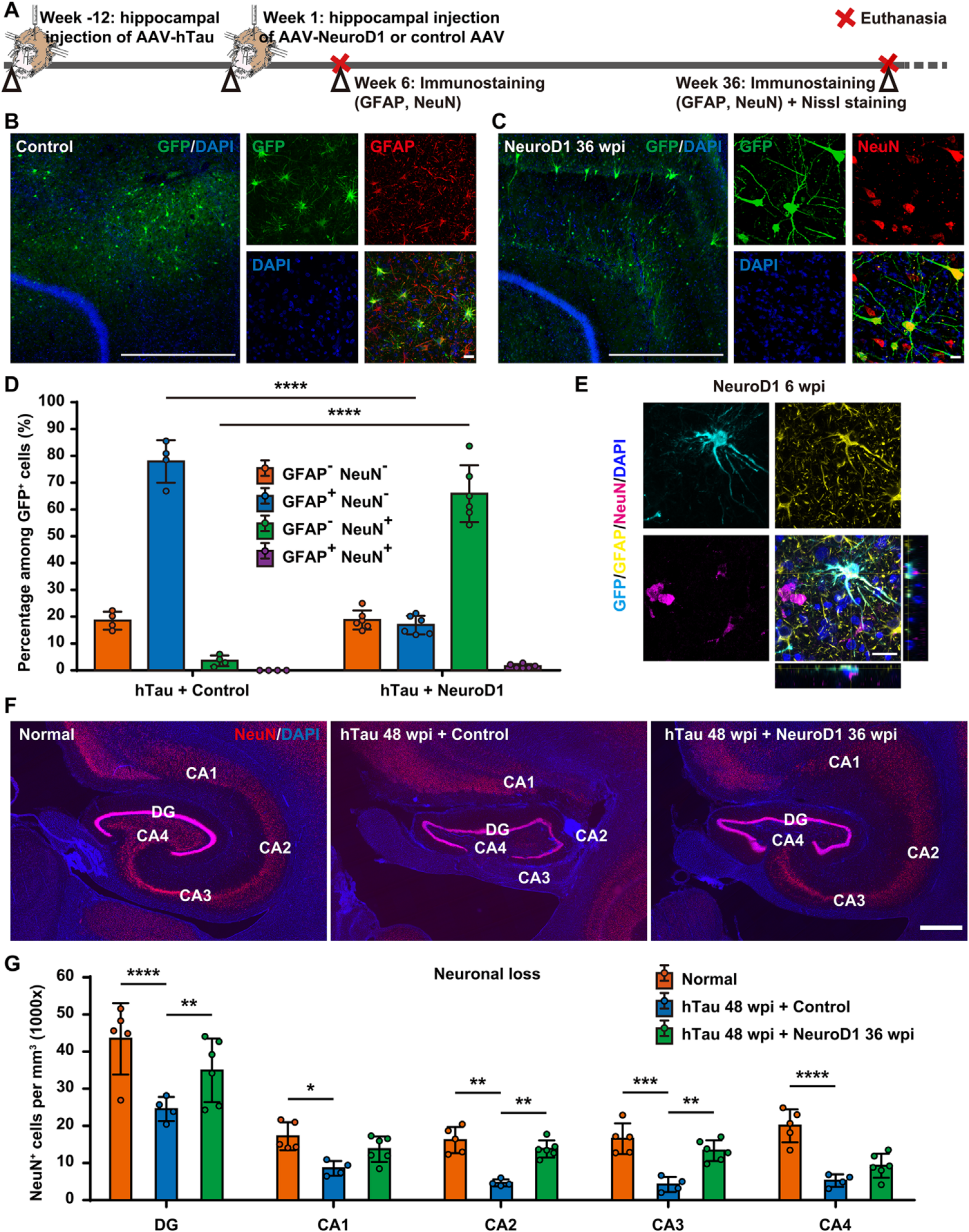
NeuroD1 AAV‐based gene therapy regenerates neurons and prevents neuronal loss in AD‐like monkeys. A) A schematic diagram depicts the process of developing the NHP AD model, administering NeuroD1 AAV‐based gene therapy, and subsequently assessing the astrocyte‐to‐neuron conversion and neuronal density in the monkey hippocampus. B) Representative images of GFP/GFAP immunostaining demonstrate that most AAV‐transduced GFP^+^ cells are GFAP^+^ astrocytes in the hippocampus of monkeys in the control group. Scale bars, 1 mm and 20 µm (inset). C) Representative images of GFP/NeuN immunostaining reveal that many AAV‐transduced GFP^+^ cells are NeuN^+^ neurons in the hippocampus of monkeys in the NeuroD1 treatment group. Scale bars, 1 mm and 20 µm (inset). D) Quantitation of the shift in cellular identity between the control group and the NeuroD1 treatment group, as indicated by the percentage of cells co‐expressing GFP with either GFAP or NeuN. ****p < 0.0001, One‐way ANOVA with Tukey's post hoc test, Control group *N* = 4, NeuroD1 group *N* = 6. E) Representative confocal images of GFP/GFAP/NeuN immunostaining with orthogonal views demonstrate potential intermediate state cells during astrocyte‐to‐neuron conversion with both astrocytic and neuronal markers. Scale bar, 20 µm. F) Representative images of NeuN immunostaining reveal a significant decrease in the number of NeuN^+^ hippocampal neurons in the control group 48 weeks after hTau expression, and a notable increase in the number of NeuN^+^ hippocampal neurons in the NeuroD1 treatment group 36 weeks after NeuroD1 expression. Scale bar, 1 mm. G) Quantification of NeuN^+^ hippocampal neuron densities to assess neuronal loss in AD‐like monkeys and evaluate neuronal regeneration/protection after NeuroD1 AAV‐based gene therapy. ****p < 0.0001, ***p < 0.001, **p < 0.01, *p < 0.05, One‐way ANOVA with Tukey's post hoc test, Normal group *N* = 5, Control group *N* = 4, NeuroD1 group *N* = 6.

Neurodegeneration is a defining feature of AD, with significant neuronal loss being observed in many rodent and NHP models of AD [18, 39]. We have previously demonstrated that NeuroD1 can convert reactive astrocytes into neurons in the cortex of 5xFAD mice [13]. Here, we further explored the neuroregenerative potential of NeuroD1 AAV‐based gene therapy in the NHP AD model (Figure 3A). Monkey hippocampal astrocytes were injected and labeled with AAV9 GFAP::GFP, along with either an AAV encoding inverted NeuroD1 (serving as a control with no functional protein expression) or an AAV encoding functional NeuroD1 as the treatment. In the control group with AAV encoding inverted NeuroD1, virtually all the GFP^+^ cells maintained astrocytic morphology, and co‐expressed astrocytic marker GFAP 36 weeks after injection (Figure 3B). In contrast, many GFP^+^ cells in the NeuroD1 treatment group had adopted neuronal morphology, and co‐expressed neuronal marker NeuN 36 weeks after infection (Figure 3C). Quantitatively, the control group consisted mainly of GFAP^+^ astrocytes (∼80%) among the GFP^+^ cells, with NeuN^+^ neurons accounting for less than 5%. Interestingly, the NeuroD1 treatment group exhibited a significant cellular identity shift, with GFAP^+^ astrocytes decreasing to ∼15% and NeuN^+^ neurons becoming the majority (∼65%) among the GFP^+^ cells, suggesting that a substantial portion of the NeuroD1‐expressing astrocytes might have been converted into neurons by 36 weeks post‐injection (Figure 3D). Meanwhile, the conversion of astrocytes into neurons was also quantified using alternative astrocytic and neuronal markers Sox9 and Tuj1. The results were consistent with Figure 3D, showing a decrease in GFP^+^ astrocytes from ∼75% to ∼15%, and an increase in GFP^+^ neurons from ∼5% to ∼60% (Figure S3, Supporting Information). Without astrocytic lineage tracing tools for NHPs, capturing an intermediate state during direct reprogramming may help confirm the astrocyte‐to‐neuron conversion in monkey hippocampus. Six weeks after NeuroD1 expression, we observed some intermediate state cells with distinct morphologies and both astrocytic and neuronal markers (GFAP and NeuN, Figure 3E), suggesting successful astrocyte‐to‐neuron conversion.

Subsequently, we conducted NeuN immunostaining to determine the neuronal density in the monkey hippocampus with or without NeuroD1 AAV‐based gene therapy. Compared to the normal monkey hippocampus (Figure 3F left), the control group with hTau overexpression followed by GFP overexpression exhibited a significant decrease in NeuN^+^ cells in the CA2/CA3/CA4 regions and a more modest reduction in the CA1/DG regions (Figure 3F middle). Conversely, NeuroD1 treatment led to a substantial restoration of NeuN^+^ cells throughout the monkey hippocampus (Figure 3F right). Quantitatively, the control group exhibited a substantial decrease in NeuN^+^ neuronal density within the hippocampal DG/CA1/CA2/CA3/CA4 regions, with a reduction of 45–75% at 48 weeks after hTau overexpression (Figure 3G). In contrast, the NeuroD1 treatment group exhibited a significant rescue in NeuN^+^ neuronal density in the same hippocampal regions compared to the control group at 36 weeks after NeuroD1 expression (Figure 3G). Considering that decreased NeuN immunoreactivity may result not only from neuronal loss but also from a depletion of the protein or a loss of its antigenicity [40], we further quantified the neuronal population with Nissl staining (cresyl violet) in the hippocampus of monkeys with or without NeuroD1 treatment. Consistent with the NeuN staining results, the Nissl‐stained cell density was notably restored in the NeuroD1 treatment group, as opposed to the control group, following a significant decrease in Nissl‐stained cell density compared to the normal monkey (Figure S4, Supporting Information). Furthermore, the GFP^+^ neurons in the monkey hippocampus were classified using markers for glutamatergic, GABAergic and cholinergic neurons. The results showed that ∼66% of GFP^+^ neurons were CTIP2^+^ glutamatergic neurons, while ∼12% were GAD67^+^ GABAergic neurons. No ChAT^+^ cholinergic GFP^+^ neurons were detected. These findings indicate that over 2/3 of GFP^+^ neurons in the monkey hippocampus, whether newly converted from astrocytes or surviving due to NeuroD1‐based intervention, are excitatory neurons, with a smaller proportion being inhibitory neurons (Figure S5, Supporting Information). Together, these results indicate that NeuroD1 AAV‐based gene therapy can effectively prevent neuronal damage and degeneration in the hippocampus of AD‐like monkeys.

### 2.4 Halting the Progression of Hippocampal Atrophy by NeuroD1 AAV‐Based Gene Therapy

**FIGURE 4:**
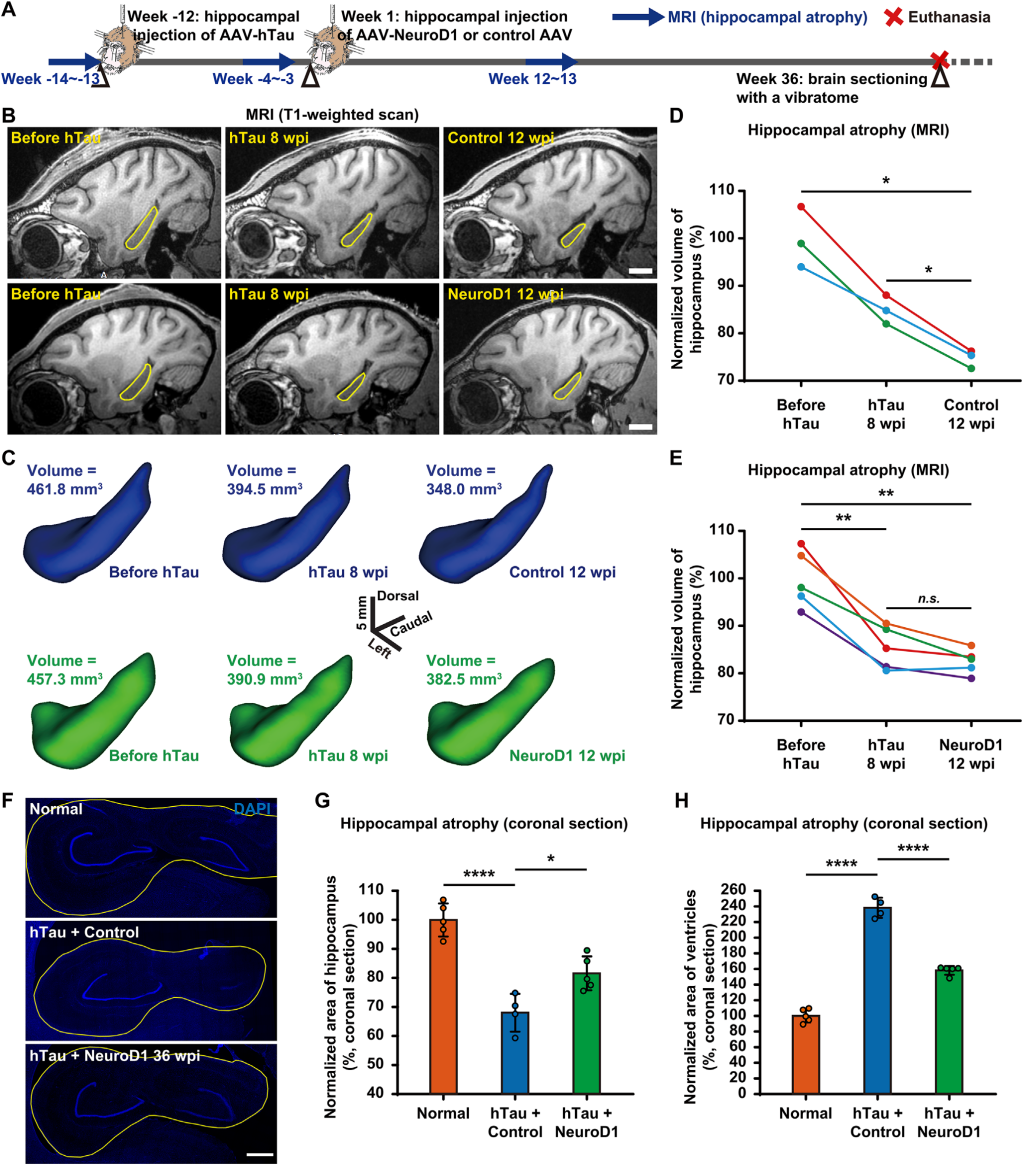
NeuroD1 AAV‐based gene therapy inhibits hippocampal atrophy in AD‐like monkeys. A) A schematic diagram illustrates the process of developing the NHP AD model, administering NeuroD1 AAV‐based gene therapy, and performing longitudinal monitoring of hippocampal volume via MRI scans and brain sectioning. B) Representative sagittal sections from T1‐weighted MRI scans reveal progressive hippocampal atrophy in NHP AD models from the control group, whereas hippocampal atrophy was halted in NHP AD models from the NeuroD1 treatment group. The yellow lines outline the boundaries of the hippocampus. Scale bar, 1 cm. C) Representative 3‐dimensional reconstructions of the monkey hippocampus obtained using the Brainsight neuronavigation system, which automatically calculates the hippocampal volume following 3‐dimensional reconstruction. Scale bar, 5 mm. D and E) Quantification of hippocampal volume changes in control group (D) or NeuroD1 treatment group (E) measured by MRI scans indicates progressive hippocampal atrophy (D) or cessation of hippocampal atrophy following NeuroD1 AAV‐based gene therapy (E). Each color represents a distinct animal. **p < 0.01, *p < 0.05, n.s., not significant, Repeated measures ANOVA with Tukey's post hoc test, *N* = 3 (D), *N* = 5 (E). F) Representative images of coronal brain sections labeled with DAPI demonstrate that NeuroD1 AAV‐based gene therapy halts the progression of hippocampal atrophy. The yellow lines delineate the boundaries of the hippocampus. Scale bar, 1 mm. G) Quantification of normalized area of hippocampus measured from coronal brain sections to assess hippocampal atrophy. ****p < 0.0001, *p < 0.05, One‐way ANOVA with Tukey's post hoc test, Normal and NeuroD1 group *N* = 5, Control group *N* = 4. H) Quantification of normalized area of ventricles measured from coronal brain sections to assess hippocampal atrophy. ****p < 0.0001, One‐way ANOVA with Tukey's post hoc test, Normal group *N* = 5, Control group *N* = 4, NeuroD1 Group *N* = 5.

Hippocampal atrophy is closely associated with cognitive impairment [35, 36]. Since NeuroD1 treatment prevented neuronal damage and degeneration in the hippocampus of NHP AD models, we further investigated whether this therapy might affect hippocampal atrophy in these AD‐like monkeys. To track changes in hippocampal volume, we employed T1‐weighted MRI brain scans before and after tau‐induced pathologies, as well as following the NeuroD1 treatment (Figure 4A). Using Brainsight neuronavigation system, we reconstructed MRI sections of the monkey hippocampi into 3D models and precisely measured their volumes at various time points following tau overexpression and the subsequent administration of AAVs encoding NeuroD1. The results revealed a steady decline in hippocampal volume within the control group, with a notable volume decrease observed 8 weeks after tau overexpression, and a further volume decrease 12 weeks after control AAV injection. However, in the NeuroD1 treatment group, the progression of hippocampal atrophy was successfully halted, with no further hippocampal shrinkage observed after NeuroD1 overexpression (Figure 4B,C, and Figure S6, Supporting Information). Quantitatively, the normalized hippocampal volumes measured from MRI scans exhibited a continuous decrease in the control group, with the shrinkage progressing from ∼15% to ∼25% over a period of 16 weeks. Conversely, in the NeuroD1 treatment group, the decline in hippocampal volume was halted after NeuroD1 overexpression, with a ∼15% volume reduction observed 8 weeks after hTau expression and no further progression following NeuroD1 expression (Figure 4D,E). To further corroborate the MRI analysis, we conducted an additional examination of hippocampal volume by comparing the mean cross‐sectional areas of the monkey hippocampus. These mean cross‐sectional areas were calculated by averaging ∼10 evenly spaced coronal brain slices, which comprehensively represent the cross‐sectional areas of the hippocampus from its anterior to middle and posterior regions. The results were consistent with those from the MRI scans, and the hippocampus of monkeys in the NeuroD1 treatment group exhibited reduced hippocampal atrophy compared to the control group, with ∼17% shrinkage as opposed to ∼30% shrinkage (Figure 4F,G). Accompanying hippocampal atrophy, the lateral ventricles of AD‐like monkeys in the control group expanded by ∼140%, whereas in the NeuroD1 treatment group, the lateral ventricles expanded by only ∼60% (Figure 4H). Taken together, these findings suggest that NeuroD1 AAV‐based gene therapy can inhibit the progression of hippocampal atrophy, presumably by preventing neuronal damage and degeneration.

### 2.5 Mitigating Neuroinflammation by NeuroD1 AAV‐Based Gene Therapy

**FIGURE 5:**
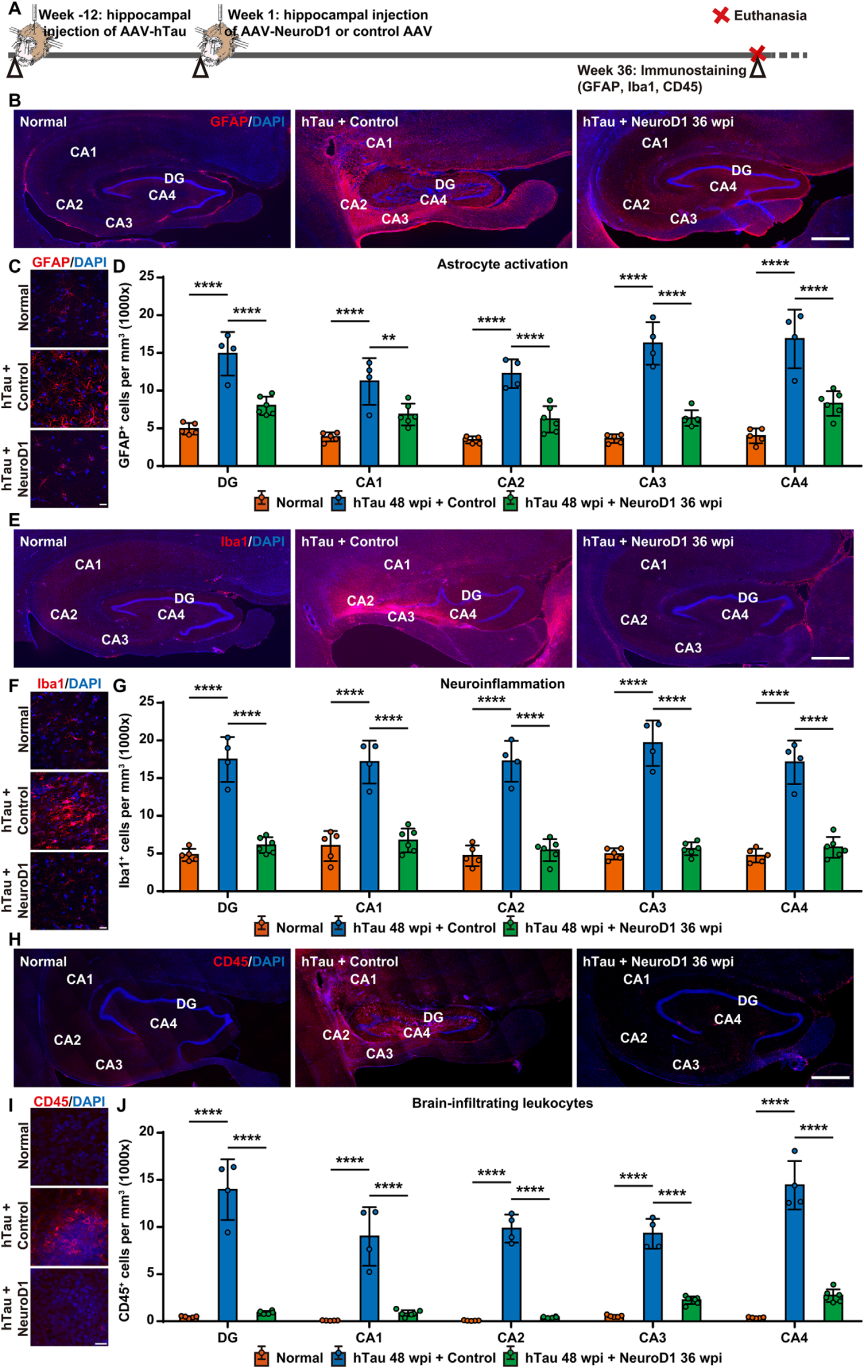
NeuroD1 AAV‐based gene therapy reduces neuroinflammation in AD‐like monkeys. A) A schematic diagram depicts the process of developing the NHP AD model, administering NeuroD1 AAV‐based gene therapy, and subsequently evaluating neuroinflammation and leukocyte infiltration in the monkey hippocampus. B) Representative images of GFAP immunostaining demonstrate astrocyte activation following hTau overexpression and a significant decrease in astrocyte activation subsequent to NeuroD1 overexpression. Scale bar, 1 mm. C) Representative images at higher magnification of GFAP immunostaining reveal the morphology of resting and reactive astrocytes in NHP hippocampus. Scale bar, 20 µm. D) Quantification of the density of GFAP^+^ cells in the hippocampus of NHP AD models. ****p < 0.0001, **p < 0.01, One‐way ANOVA with Tukey's post hoc test, Normal group *N* = 5, Control group *N* = 4, NeuroD1 group *N* = 6. E) Representative images of Iba1 immunostaining demonstrate microglia activation following hTau overexpression and a significant decrease in microglia activation subsequent to NeuroD1 overexpression. Scale bar, 1 mm. F) Representative images at higher magnification of Iba1 immunostaining reveal the morphology of resting and activated microglia in NHP hippocampus. Scale bar, 20 µm. G) Quantification of the density of Iba1^+^ cells in the hippocampus of NHP AD models. ****p < 0.0001, One‐way ANOVA with Tukey's post hoc test, Normal group *N* = 5, Control group *N* = 4, NeuroD1 group *N* = 6. H) Representative images of CD45 immunostaining demonstrate leukocyte infiltration following hTau overexpression and a significant decrease in leukocyte infiltration subsequent to NeuroD1 overexpression. Scale bar, 1 mm. I) Representative images at higher magnification of CD45 immunostaining reveal the morphology of brain‐infiltrating leukocytes in NHP hippocampus. Scale bar, 20 µm. J) Quantification of the density of CD45^+^ cells in the hippocampus of NHP AD models. ****p < 0.0001, One‐way ANOVA with Tukey's post hoc test, Normal group *N* = 5, Control group *N* = 4, NeuroD1 group *N* = 6.

Neuroinflammation, mediated by cells like microglia and astrocytes, has been proposed as a key mechanism for AD development, ultimately leading to neuronal death and dysfunction [41, 42]. GFAP is a well‐established marker of astrocyte activation, and changes in its expression level often correlate with neuroinflammatory processes. We carried out GFAP and Sox9 immunostaining (Figure 5A) and found excessive astrocyte activation in the hippocampus of AD‐like monkeys from the control group, but not from the NeuroD1 treatment group at 36 weeks after gene therapy (Figure 5B and Figure S7, Supporting Information). Enlarged images showed that the GFAP^+^ cells in the control group exhibited typical reactive astrocyte morphology, such as cytoskeletal hypertrophy and outgrowth of exceptionally long processes. In contrast, the astrocytes in the NeuroD1 treatment group closely resembled the morphology of resting astrocytes found in the hippocampus of healthy monkey (Figure 5C). Quantitatively, the number of GFAP^+^ and Sox9^+^ cells in the hippocampus of monkeys from the control group exhibited a significant increase, ranging from 3 to 4 times the normal level. However, in the NeuroD1 treatment group, there was a notable decrease in the number of GFAP^+^ and Sox9^+^ cells in the hippocampus compared to the control group, suggesting that NeuroD1 AAV‐based gene therapy can effectively reduce astrocyte reactivity in the hippocampus of NHP AD models (Figure 5D and Figure S7, Supporting Information).

Microglial activation is another defining feature of neuroinflammation associated with AD [41, 43, 44]. We conducted Iba1 immunostaining to assess the activation of microglia (Figure 5A) and found that the number of Iba1^+^ cells increased dramatically in the control group compared to the normal level, whereas in the NeuroD1 treatment group there was a substantial reduction in the number of Iba1^+^ cells (Figure 5E). Even more intriguingly, within the hippocampi of monkeys in the NeuroD1 treatment group, most Iba1^+^ cells regained the ramified, star‐shaped appearance of resting microglia. Conversely, in the control group, a considerable proportion of Iba1^+^ cells underwent morphological changes, adopting small, spherical, rod‐shaped, or amoeboid‐like morphologies of activated microglia (Figure 5F). Quantitatively, the density of Iba1^+^ cells exhibited a substantial increase in all regions of the hippocampus in the control group, rising 3 to 4 times above baseline. In contrast, the density of Iba1^+^ cells dropped significantly in the NeuroD1 treatment group, with an elevation of 15–30% compared to baseline, indicating that NeuroD1 AAV‐based gene therapy can effectively diminish microglia activation in the hippocampus of AD‐like monkeys (Figure 5G).

Brain‐infiltrating leukocytes and macrophages are thought to contribute to the progression of AD by promoting blood‐brain barrier (BBB) impairment, elevating chronic inflammatory response, and exacerbating neuronal damage [45, 46, 47]. CD45 is a pan‐leukocyte marker [48], and we performed CD45 staining in the hippocampi of NHP AD models 36 weeks post‐injection of either control or NeuroD1‐expressing AAVs (Figure 5A). While CD45 labels leukocytes, macrophages and microglia within the CNS, our findings suggest that the majority of CD45^+^ cells in our monkey brain slices do not express Iba1, indicating that most of these CD45^+^ cells are unlikely to be microglia (Figure S8, Supporting Information). In fact, there were almost no detectable CD45^+^ leukocytes and macrophages in the hippocampus of healthy monkeys, whereas AD‐like monkeys in the control group exhibited a massive presence of CD45^+^ leukocytes and macrophages. However, the NeuroD1 treatment group showed a significant reduction in CD45 signal levels, bringing them close to those observed in healthy monkeys (Figure 5H,I). Quantitatively, the number of CD45^+^ cells increased by at least 10 times in every region of the hippocampus following hTau expression, whereas it decreased almost to the level observed in the healthy monkey 36 weeks after NeuroD1 expression (Figure 5J). Collectively, these findings suggest that NeuroD1 AAV‐based gene therapy can successfully alleviate neuroinflammation in the hippocampus of AD‐like monkeys.

### 2.6 Restoring Vascular and BBB Integrity by NeuroD1 AAV‐Based Gene Therapy

**FIGURE 6:**
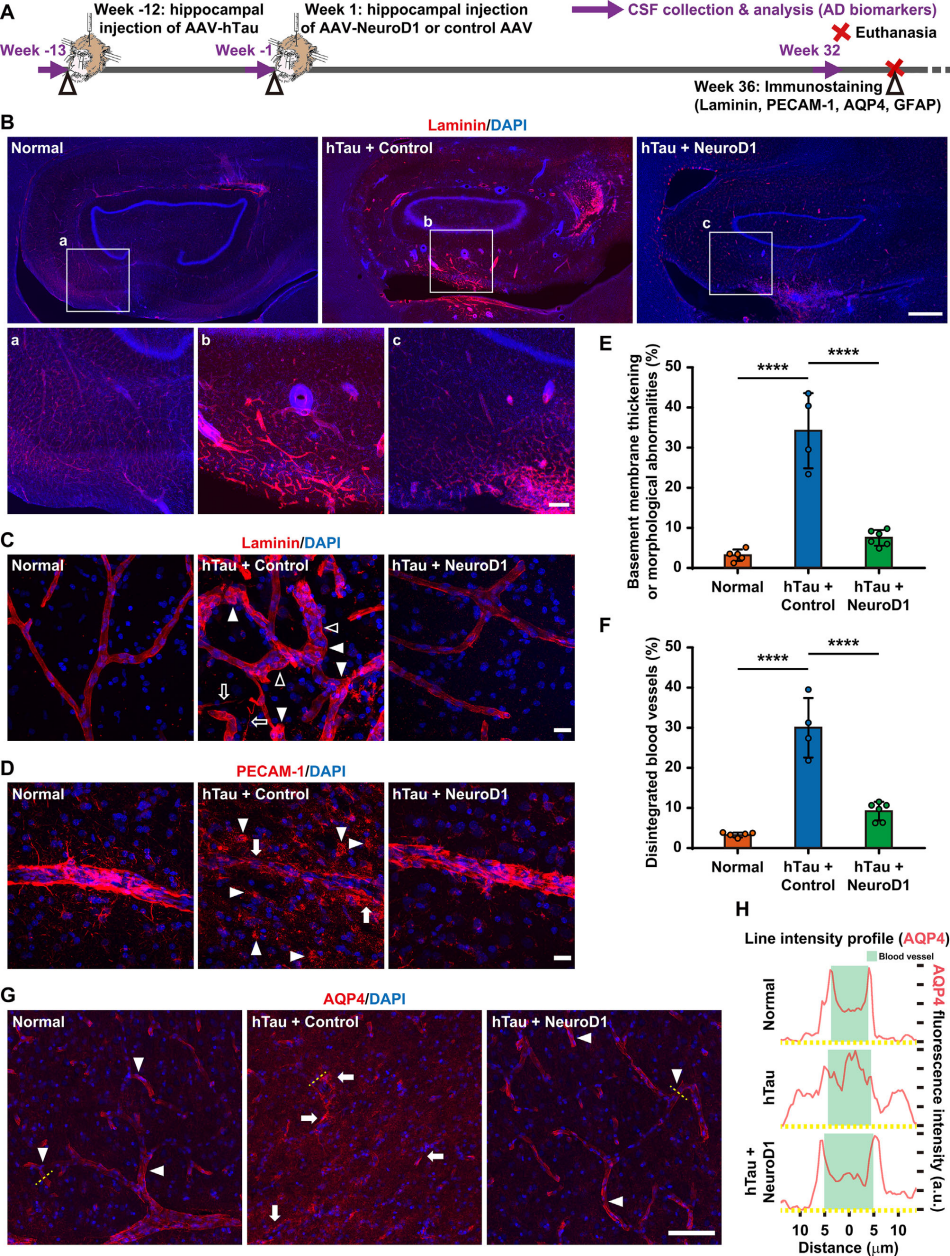
NeuroD1 AAV‐based gene therapy repairs vascular and BBB damage in AD‐like monkeys. A) A schematic diagram depicts the development of the NHP AD model, followed by NeuroD1 AAV‐based gene therapy, evaluation of vascular/BBB integrity in monkey hippocampus, and longitudinal monitoring of AD biomarkers in CSF using Simoa. B and C) Representative images of laminin immunostaining demonstrate basement membrane thickening, vascular damage (B), and morphological abnormalities of the blood vessels (C, at higher magnification) in the control group. Note that the staining pattern in the NeuroD1 treatment group resembles that observed in healthy monkeys. Arrows, string vessels; open arrowheads, vascular occlusion; closed arrowheads, tortuous/bulging vessels. Scale bars, 1 mm (B), 200 µm (B, inset), and 20 µm (C). D) Representative images at higher magnification of PECAM‐1 immunostaining reveal vascular damage and degeneration in the control group. Note that the staining pattern in the NeuroD1 treatment group resembles that observed in healthy monkeys. Arrows, blood vessel ruptures; arrowheads, blood vessel disintegration. Scale bar, 20 µm. E and F) Quantification of the percentage of blood vessels exhibiting basement membrane thickening, morphological abnormalities (E), and vascular degeneration (F). ****p < 0.0001, One‐way ANOVA with Tukey's post hoc test, Normal group *N* = 5, Control group *N* = 4, NeuroD1 Group *N* = 6. G) Representative images of AQP4 immunostaining reveal the distribution patterns of AQP4. Note the polarized distribution of AQP4 surrounding blood vessels in the normal and NeuroD1 treatment group, and the dispersed distribution of AQP4 in the control group. Arrows, dispersed signals; arrowheads, polarized signals. Scale bar, 100 µm. H) Representative line‐scan intensity profiles (pink lines) measured along line segments perpendicular to blood vessels. The turquoise block indicates the width of a specific blood vessel. Line segment, 40 µm.

Vascular issues, including chronic brain hypoperfusion, BBB leakage, and persistent vascular inflammation, play significant roles in the progression of sporadic AD [49, 50]. The vascular basement membrane thickens in neurodegenerative disorders, potentially impeding blood flow to the brain and accelerating disease progression [51]. We performed staining for laminin, a basement membrane marker (Figure 6A), and observed vascular basement membrane thickening and vascular abnormalities in the control group (Figure 6B,C). The abnormalities included string vessels (open arrows), vascular occlusion (open arrowheads), and tortuous/bulging vessels (closed arrowheads, Figure 6C middle). Conversely, the hippocampal blood vessels exhibited a less thickened morphology and significantly fewer abnormalities in the NeuroD1 treatment group (Figure 6B,C right). Furthermore, we conducted staining for PECAM‐1/CD31, an endothelial marker (Figure 6A), and observed notable vascular damage/degeneration, marked by blood vessel ruptures (arrows) and disintegration (arrowheads) in the control group (Figure 6D middle). In contrast, the hippocampal blood vessels showed much less vascular damage/degeneration in the NeuroD1 treatment group (Figure 6D right). Quantitatively, basement membrane thickening, vascular damage and degeneration increased by ∼8–12 times in the control group, whereas they only increased by ∼2–3 times in the NeuroD1 treatment group, indicating that NeuroD1 AAV‐based gene therapy has the potential to repair vascular damage in the hippocampus of NHP AD models (Figure 6E,F).

AQP4 is predominantly expressed in astrocyte endfeet, which are crucial for the formation of the BBB, and disruption in AQP4 function can lead to BBB breakdown [52]. In the glymphatic system, AQP4 enables the efficient exchange of water and solutes between the CSF and interstitial fluid of the brain, thereby facilitating the clearance of waste products from the brain [53, 54]. AQP4 immunostaining showed mis‐localized signals (arrows) in the control group (Figure 6G middle). Conversely, the distribution of AQP4 signal was largely restored (arrowheads), and AQP4 was detected surrounding blood vessels in the normal and NeuroD1 treatment groups (Figure 6G left right). The AQP4 distribution was quantified using line‐scan intensity profiles perpendicular to blood vessels (yellow dashed lines, Figure 6G). We observed a dispersed distribution of AQP4 in the control group, and a polarized distribution in the normal and NeuroD1 treatment groups (Figure 6H), suggesting that hTau overexpression disrupts BBB integrity, which can be partially restored via NeuroD1 AAV‐based gene therapy.

Total tau, phospho‐tau 181, and phospho‐tau 231 in the CSF are positively correlated with the severity of AD, while Aβ42 peptide and Aβ42/Aβ40 ratio in the CSF are negatively correlated, making them specific biomarkers for AD [55, 56, 57]. Furthermore, mis‐localization of astrocytic AQP4 can disrupt the perivascular drainage and glymphatic system, ultimately leading to Aβ/tau clearance deficit [54, 58, 59]. We utilized the Simoa technique to measure total tau, phospho‐tau 181, and phospho‐tau 231 in the monkey CSF samples collected before and after 12 weeks of hTau overexpression, as well as after 32 weeks of NeuroD1 overexpression. Total tau, phospho‐tau 181, and phospho‐tau 231 were significantly elevated after 12 weeks of hTau overexpression, but decreased notably after 32 weeks of NeuroD1 expression (Figure S9A,B, Supporting Information). Similarly, we assessed Aβ42, Aβ40, and Aβ42/Aβ40 ratio in the monkey CSF samples, finding a significant decrease in Aβ42 levels and the Aβ42/Aβ40 ratio following 12 weeks of hTau overexpression. However, after 32 weeks of NeuroD1 expression, both Aβ42 levels and the Aβ42/Aβ40 ratio significantly increased, indicating enhanced Aβ clearance after NeuroD1 treatment (Figure S9C,D, Supporting Information). Together, NeuroD1 AAV‐based gene therapy can effectively repair vascular and BBB damages, and potentially help waste clearance in AD‐like monkeys.

### 2.7 Improving Hippocampal Glucose Metabolism and Spatial Working Memory by NeuroD1 AAV‐Based Gene Therapy

**FIGURE 7:**
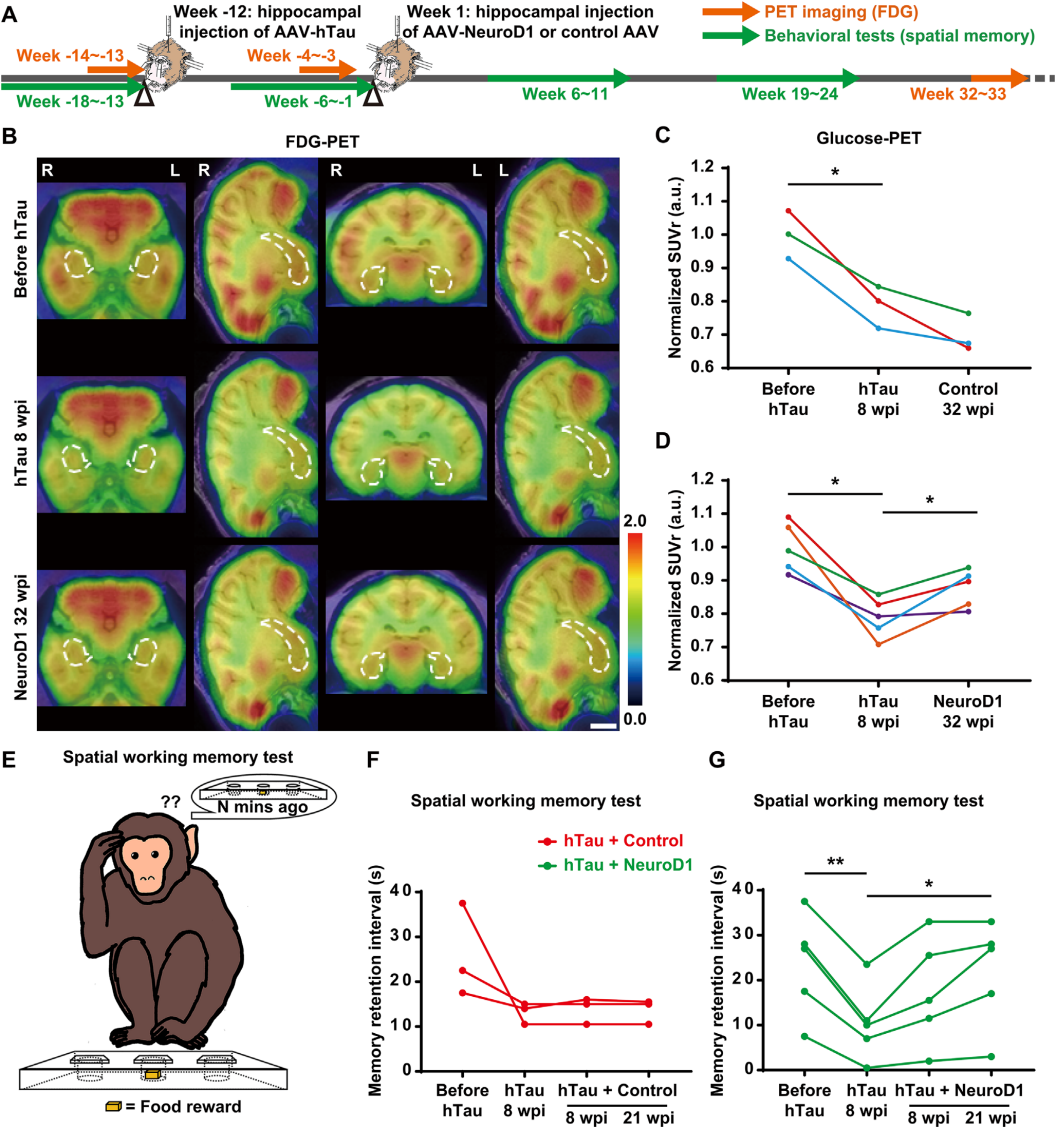
NeuroD1 AAV‐based gene therapy enhances glucose metabolism and spatial memory in AD‐like monkeys. A) A schematic diagram illustrates the process of developing the NHP AD model, administering NeuroD1 AAV‐based gene therapy, and conducting longitudinal monitoring of hippocampal glucose metabolism and spatial working memory using FDG PET scans and NHP behavioral tests. B) Representative ^18^F‐FDG PET/MRI fusion images demonstrate reduced hippocampal glucose metabolism after 8 weeks of hTau overexpression, and increased hippocampal glucose metabolism 32 weeks after NeuroD1 expression. The SUV color scale is in the bottom right corner. Dashed lines outline the hippocampal boundaries. Scale bar, 1 cm. C and D) Quantification of normalized ^18^F‐FDG retention in the hippocampus of monkeys from the control group (C) and the NeuroD1 treatment group (D). Each color represents a distinct animal. *p < 0.05, Repeated measures ANOVA with Tukey's post hoc test, *N* = 3 (C), *N* = 5 (D). E) A schematic diagram depicts the “delayed response” task, utilizing the WGTA to assess the “memory retention interval” and thereby evaluate the spatial working memory capacity of the NHPs before and after hTau overexpression, as well as following NeuroD1 AAV‐based gene therapy. F and G) Quantitation of the “memory retention interval” recorded in the monkeys from the control group (F) and the NeuroD1 treatment group (G). Each polyline represents a distinct animal. **p < 0.01, *p < 0.05, Repeated measures ANOVA with Tukey's post hoc test, *N* = 3 (F), *N* = 5 (G).

Fluorine‐18 fludeoxyglucose (^18^F‐FDG) PET scan is a sensitive method for identifying alterations in glucose metabolism in the brain. Notably, reduced metabolic activity in specific brain regions strongly correlates with the pathological diagnosis of AD [60, 61], and effective AD treatments tend to result in improved metabolic activity in the hippocampus [62, 63]. Hence, we conducted longitudinal ^18^F‐FDG PET brain imaging on the same group of monkeys, assessing them before and after hTau overexpression, as well as following NeuroD1 AAV‐based gene therapy (Figure 7A). Consistent with the neuronal loss depicted in Figure 3, the monkey hippocampus exhibited a notable decrease in glucose metabolism after 8 weeks of hTau overexpression, with a further decline at 32 weeks post AAV injection in the control group (Figure 7B,C). In contrast, following 32 weeks of NeuroD1 overexpression, a significant improvement in glucose metabolism was observed within the hippocampus of AD‐like monkeys in the NeuroD1 treatment group, indicating the restoration of neuronal activity following NeuroD1 AAV‐based gene therapy (Figure 7B,D). Quantitatively, there was a significant decrease in glucose metabolism after 8 weeks of hTau overexpression, indicating considerable neuronal damage and degeneration in the monkey hippocampus. Subsequently, glucose metabolism continued to decline in the control group 32 weeks after AAV injection (Figure 7C), suggesting further neuronal damage and loss. In contrast, following 32 weeks of NeuroD1 AAV‐based gene therapy, there was a partial yet substantial recovery in glucose metabolism in the hippocampus of AD‐like monkeys (Figure 7D), suggesting a potential restoration of neuronal function in the NeuroD1 treatment group.

Spatial working memory are crucial for comprehending the progression of AD pathology and assessing potential therapeutic interventions for AD [34, 64]. Although working memory processes have traditionally been associated with the prefrontal cortex, many studies have provided substantial evidence of hippocampal involvement in spatial working memory [65, 66]. To evaluate the spatial working memory of rhesus monkeys, a “delayed response” (DR) task using the Wisconsin General Test Apparatus (WGTA) has been generally employed to gain valuable insights into the cognitive abilities of non‐human primates [67, 68]. In the DR task, monkeys were required to briefly memorize the location of food rewards concealed in one of three covered wells. A monkey was considered to have reliably memorized the location of the food rewards if, following a designated delay period with its vision obscured by an opaque screen, it accurately selected the baited well on the first attempt in at least 26 out of 30 daily trials over 3 consecutive days. The “memory retention interval” refers to the maximum delay duration during which a monkey could consistently retain the spatial information in its working memory in this task (Figure 7E and Movie S1, Supporting Information). After 8 weeks of hTau expression, the monkeys exhibited a substantial decline in their “memory retention interval”, indicating a significant impairment in spatial working memory capacity (Figure 7F,G). Eight to twenty‐one weeks after AAV injection, the monkeys in the control group maintained a low “memory retention interval” (Figure 7F). Conversely, the monkeys in the NeuroD1 treatment group showed a remarkable improvement in their “memory retention interval” within the same time window (Figure 7G). To evaluate the associative learning ability of rhesus monkeys, we also performed the “delayed match‐to‐sample” (DMTS) and “delayed nonmatch‐to‐sample” (DNMTS) tasks on AD‐like monkeys treated with NeuroD1 AAV‐based gene therapy. The results showed a trend toward partial recovery of associative learning ability following gene therapy, though it did not reach statistical significance, likely due to inter‐animal variability and a limited sample size compared to rodent behavioral studies (Figure S10, Supporting Information). These results provide compelling evidence that NeuroD1 AAV‐based gene therapy can effectively ameliorate deficits in spatial working memory in AD‐like monkeys.

### 2.8 Upregulating Neuronal Function and Synaptic Transmission and Downregulating Neuroinflammation and Apoptosis by NeuroD1 Revealed by Transcriptome Analysis

**FIGURE 8:**
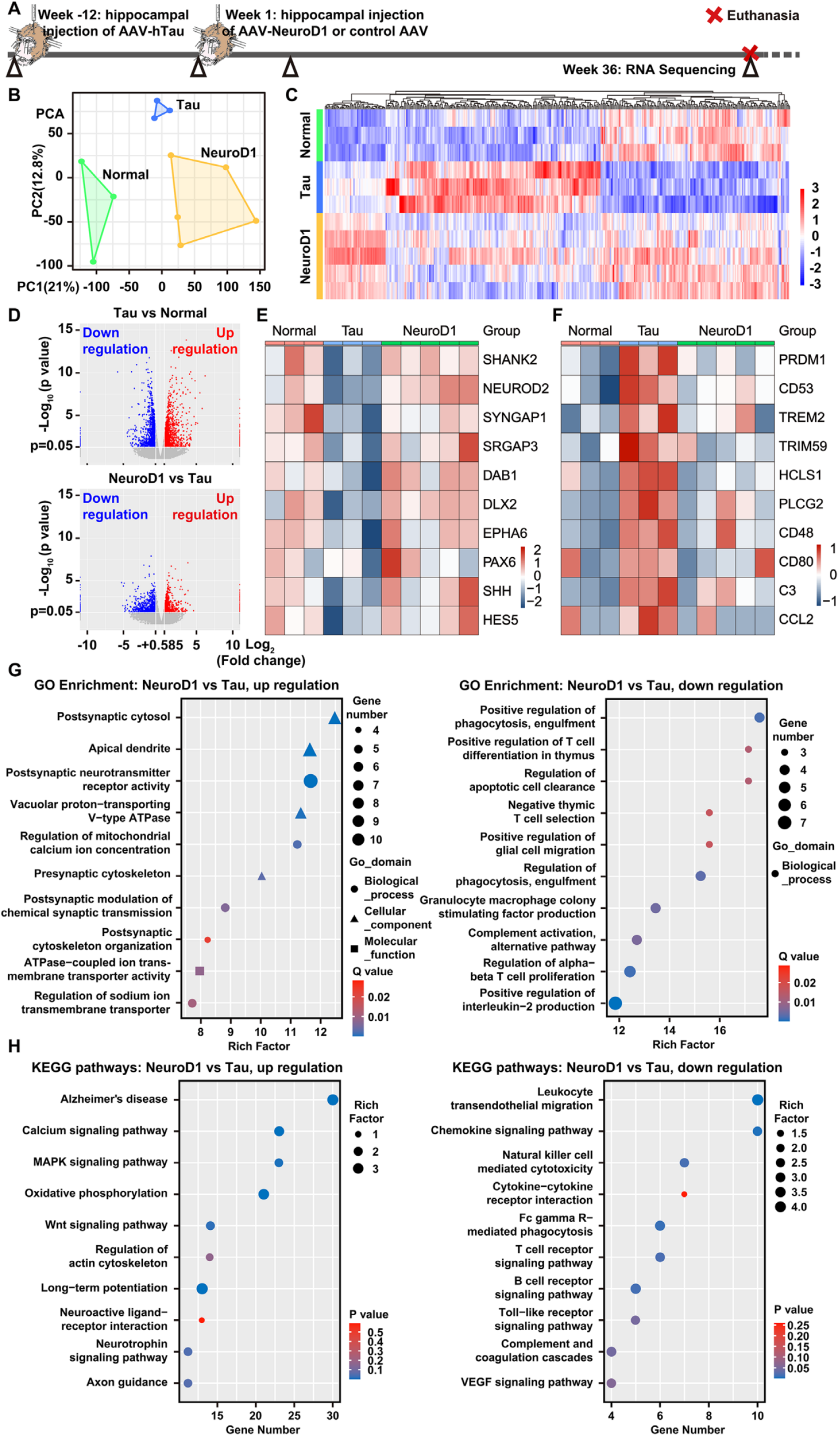
Upregulation of neuronal function and synaptic transmission and downregulation of neuroinflammation and apoptosis by NeuroD1 revealed by transcriptome analysis. A) A schematic diagram illustrates the process of developing the NHP AD model, administering NeuroD1 AAV‐based gene therapy, and subsequently performing bulk RNA sequencing to conduct transcriptome analysis. B) PCA dimension reduction plot of the bulk RNA sequencing data. Please note the good intra‐group consistency of the transcriptome data of monkeys in the normal (green), hTau‐overexpressing control (blue), and NeuroD1 treatment groups (yellow). C) Heatmap of 400+ DEGs among the normal (green), hTau‐overexpressing control (blue), and NeuroD1 treatment groups (yellow) with hierarchical clustering. Color scale (bottom right) indicates the degree of expression: red, high expression; blue, low expression. The selection criteria for DEGs were *Q*‐value ≤ 0.05, and |log_2_FC| ≥ 1. D) Volcano plots of DEGs. X axis represents log_2_ transformed fold change. Y axis represents −log_10_ transformed significance. Red points, upregulated genes; blue points, downregulated genes. Top, hTau‐overexpressing control group versus normal group; bottom, NeuroD1 treatment group versus hTau‐overexpressing control group. E) Heatmap of top 10 DEGs that were downregulated in the AD‐like monkeys and subsequently upregulated after the gene therapy. Color scale (bottom right) indicates the degree of expression: red, high expression; blue, low expression. F) Heatmap of top 10 DEGs that were upregulated in the AD‐like monkeys and subsequently downregulated after the gene therapy. Color scale (bottom right) indicates the degree of expression: red, high expression; blue, low expression. G) Bubble plots of GO enrichment analysis of DEGs. Top 10 GO terms are enriched. Rich factor demonstrates the degree of enrichment by GO. The node size represents the number of selected genes, and color represents the *Q* value of the enrichment analysis. *Q* value is a multiple hypothesis‐corrected *p* value. H) Bubble plots of KEGG pathway analysis of DEGs. Top 10 KEGG pathways are enriched. The node size represents Rich factor, and color represents the *p* value of the enrichment analysis. Rich factor is the ratio of the number of DEGs annotated in a pathway (as indicated in the *y*‐axis) to the number of all genes annotated in this pathway.

Transcriptome analysis is a potent tool capable of identifying gene expression changes, elucidating molecular mechanisms and pathways involved in diseases or biological functions, guiding therapeutic strategies by disclosing treatment effects, and uncovering novel gene functions and their potential contributions to health and disease [69, 70]. Therefore, we conducted RNA sequencing and transcriptome analysis to identify differentially expressed genes (DEGs) and signaling pathways modulated by NeuroD1 overexpression to attempt to decipher the molecular mechanisms of NeuroD1 AAV‐based gene therapy (Figure 8A). Principal Component Analysis (PCA) revealed that the transcriptome data of 3 normal monkeys, 3 hTau‐overexpressing AD‐like monkeys, and 5 AD‐like monkeys treated with NeuroD1 AAV‐based gene therapy were clustered into three discrete regions in the PCA dimension reduction plot, indicating intra‐group consistency of the transcriptome data (Figure 8B). The heatmap of DEGs with hierarchical clustering not only demonstrated data consistency within groups, but also displayed comprehensive patterns and relationships of 400+ DEGs among different groups. Many DEGs upregulated in the control group were downregulated in the NeuroD1 treatment group, and vice versa, suggesting that NeuroD1 AAV‐based gene therapy systematically restored the brains of AD‐like monkeys to a healthier state (Figure 8C). Volcano plots of DEGs showed 1579 genes upregulated and 1353 genes downregulated (*p* < 0.05) between the hTau‐overexpressing control and normal groups (Figure 8D upper), as well as 974 genes upregulated and 745 genes downregulated between the NeuroD1 treatment and hTau‐overexpressing control groups (Figure 8D lower).

All these DEGs were categorized to identify those that were downregulated in the AD‐like monkeys and subsequently upregulated after gene therapy, as well as those that were upregulated in the AD‐like monkeys and downregulated post‐treatment. The former group, including *SHANK2*, *NEUROD2*, *SYNGAP1*, *SRGAP3*, *DAB1*, *DLX2*, *EPHA6*, *PAX6*, *SHH*, and *HES5*, is implicated in neurogenesis, neuronal differentiation and migration, and synaptic structure and function (Figure 8E). The latter group, including *PRDM1*, *CD53*, *TREM2*, *TRIM59*, *HCLS1*, *PLCG2*, *CD48*, *CD80*, *C3*, and *CCL2*, is associated with inflammation, microglial and leukocyte function, innate immune response and phagocytosis, as well as immune cell activation and antigen presentation (Figure 8F). These findings highlight the dual role of NeuroD1 AAV‐based gene therapy in promoting neuroregeneration/neuroprotection while attenuating neuroinflammatory responses.

Gene Ontology (GO) enrichment and Kyoto Encyclopedia of Genes and Genomes (KEGG) pathway analysis identify biological processes, molecular functions, and cellular components associated with a set of genes, and map genes to known signaling and metabolic pathways involved in diseases or biological processes [71, 72, 73]. Thus, we performed GO enrichment analysis of DEGs, and it revealed that in the hippocampus of AD‐like monkeys, biological processes related to neuroinflammation, apoptosis, autophagy, synapse pruning, cytotoxicity, and amyloid fibril formation were significantly upregulated; whereas the functions of ion channels and transporters, presynaptic neurotransmitter release, nuclear RNA surveillance, and postsynaptic neurotransmitter receptor transport were significantly downregulated (Figure S11A, Supporting Information). Similarly, KEGG pathway analysis of DEGs demonstrated that in the hippocampus of AD‐like monkeys, signaling pathways related to Alzheimer's disease, many other neurodegenerative diseases, neuroinflammation, phagocytosis, and innate immune responses were significantly upregulated; whereas major neurodevelopmental signaling pathways, neuroactive ligand‐receptor interaction, and metabolic regulation signaling pathways were significantly downregulated (Figure S11B, Supporting Information).

Opposite to the transcriptome changes induced by hTau overexpression, in the hippocampus of NHPs in the NeuroD1 treatment group, GO enrichment analysis of DEGs revealed that biological processes and cellular components related to pre‐ and postsynaptic assembly, apical dendrite, mitochondrial energy metabolism, resting membrane potential maintenance, and synaptic transmission were significantly upregulated; whereas biological processes related to neuroinflammatory responses, apoptosis, and phagocytosis were significantly downregulated (Figure 8G). Similarly, KEGG pathway analysis of DEGs demonstrated that in the hippocampus of NHPs in the NeuroD1 treatment group, signaling pathways related to neurodevelopment, calcium signaling, mitochondrial energy metabolism, long‐term potentiation, axon guidance, and neuroactive ligand–receptor interaction were significantly upregulated; whereas signaling pathways related to pathological neuroinflammation, innate immune responses, phagocytosis, and the formation of new blood vessels were significantly downregulated (Figure 8H). Taken together, transcriptome analysis indicates that NeuroD1 AAV‐based gene therapy can systematically restore the brains of AD‐like monkeys to a healthier state, primarily by upregulating neuronal function and synaptic transmission, while downregulating neuroinflammation and apoptosis.

## 3 Discussion

In this study, AAVs encoding NeuroD1 were delivered to the hippocampus of AD‐like monkeys. The widespread expression of NeuroD1 across the hippocampus prevented neuronal degeneration, halted hippocampal atrophy, reduced neuroinflammation, and repaired vascular and BBB damages. Additionally, CSF AD biomarkers, hippocampal metabolism, and spatial working memory were all partially restored. Transcriptome analysis indicated that NeuroD1 AAV‐based gene therapy upregulated neuronal development and synaptic transmission, and downregulated neuroinflammation and apoptosis, presenting a promising multi‐faceted approach for AD treatment.

Currently existing treatments for AD mainly focus on symptom management and slowing disease progression. Cholinesterase inhibitors and NMDA antagonist offer modest cognitive benefits but do not alter the disease's progression. Disease‐modifying treatments like anti‐amyloid antibodies can reduce amyloid plaques, but their impact on cognition and function is limited, with long‐term efficacy and safety still under scrutiny. Additionally, delivering drugs to the CNS remains challenging due to the BBB, and the long‐term safety of new treatments remains a concern. Ongoing research is focusing on developing disease‐modifying therapies targeting beyond amyloid‐β and tau, such as neuroregeneration, neuroinflammation, BBB, mitochondrial dysfunction, and exploring innovative drug delivery methods, such as nanotechnology and intranasal administration [74, 75, 76]. In response to the limitations of current AD treatments, we developed NeuroD1 AAV‐based gene therapy to address the challenge of repairing and replacing damaged neurons. Encouragingly, this therapy prevented neuronal damage and loss in NHP AD models, upregulated neurodevelopment and synaptic transmission, and downregulated apoptosis. Additionally, it alleviated neuroinflammation, repaired the BBB, and reduced VEGF‐induced angiogenesis. Thus, this innovative approach targets neuroregeneration, neuroinflammation and the BBB, delivering widespread NeuroD1 throughout the monkey hippocampus via a single stereotactic injection.

The NeuroD1 AAV‐based gene therapy demonstrates therapeutic potential by mitigating neuroinflammation and restoring BBB integrity. Ectopic NeuroD1 expression reprograms reactive A1 astrocytes, curtailing the release of pro‐inflammatory cytokines such as TNF‐α, IL‐1β, and IL‐18, while attenuating microglial activation and shifting microglia toward a less neurotoxic state. This fosters a neuroprotective microenvironment, evidenced by elevated PTX3 expression in A2 astrocytes. Particularly, NeuroD1 promotes BBB repair through multifaceted mechanisms. First, NeuroD1 may attenuate reactive astrocyte‐mediated neuroinflammation by polarizing astrocytes toward an anti‐inflammatory A2 phenotype, reducing the release of pro‐inflammatory cytokines and mitigating BBB disruption. Second, NeuroD1 overexpression re‐establishes astrocytic endfeet‐AQP4 associations with vasculature, reducing BBB permeability and leakage. Third, NeuroD1 upregulates endothelial tight junction proteins ZO‐1 and occludin via modulation of genes like *Ier5* and *Rn7sk*, countering endothelial damage. Finally, the promotion of neurotrophic factors such as pleiotrophin (PTN) by NeuroD1 contributes to the improvement of cellular microenvironment, further supporting endothelial stability and BBB integrity [77, 78, 79].

Transgenic mouse models have driven the development of AD treatments currently in clinical trials, including gene therapies for neurogenesis and neuroprotection, Aβ and tau‐targeted therapies, neuroinflammation modulation, BBB repair, and stem cell‐based therapies. However, their limitations, such as short lifespan, incomplete disease recapitulation, translational and delivery challenges, side effects, and lack of endpoint standardization and translatable biomarkers, highlight why successful interventions in mice often fail to translate to patients [80, 81, 82]. Glia‐to‐neuron conversion shows promise for treating AD by addressing both neuronal loss and neuroinflammation. Reactive glial cells in the mouse cortex can be directly reprogrammed into functional neurons in vivo using transcription factors like NeuroD1, offering a potential avenue for treating AD. While early studies in mice show encouraging results, further research is needed to evaluate the safety and efficiency of this approach before potential clinic trials [13, 83]. To assess whether the transcription factor NeuroD1, which generates functional neurons in AD mouse models, holds potential for treating human AD, we tested NeuroD1 overexpression in an NHP AD model in this study. Given their close genetic, anatomical, and physiological similarities to humans, NHPs offer a more accurate model for evaluating the therapeutic potential of NeuroD1 AAV‐based gene therapy in repairing brain structure and function in AD patients.

Many pathological studies of AD indicate that it originates in the entorhinal cortex and hippocampus. Based on this, we initially overexpressed hTau in the hippocampus of rhesus monkeys to develop an AD‐like NHP model [18]. We also overexpressed NeuroD1 in the hippocampus to assess the safety and efficacy of NeuroD1 AAV‐based gene therapy. However, AD ultimately leads to widespread brain damage. Therefore, an optimal NHP AD model should exhibit key AD pathological features not just in the hippocampus but throughout the entire brain. Thus, a comprehensive evaluation of the safety and efficacy of NeuroD1 AAV‐based gene therapy should ideally be conducted across the entire brains of NHP models with AD‐like pathologies, which represents an important direction for future investigation.

The 2024 expert consensus (spearheaded by both international and Chinese guidelines) has formally integrated Tau pathology burden as a biological criterion for staging AD severity [84]. Therefore, we evaluated whether NeuroD1 overexpression affect Tau pathology by performing immunostaining against Tau and phospho‐Tau (pTau) in the hippocampus of normal monkeys, AD‐like monkeys, and AD‐like monkeys treated with NeuroD1 AAV‐based gene therapy. The results showed no significant attenuation of Tau pathology after NeuroD1 overexpression, which may be attributable to the persistent AAV‐driven hTau overexpression (Figure S12, Supporting Information). However, both Tau and pTau exhibited subtle decreasing trends, potentially associated with enhanced CSF waste clearance mediated by NeuroD1 AAV‐based gene therapy (Figure S12, Supporting Information).

A growing body of evidence has demonstrated notable sex differences in both the epidemiology and pathological mechanisms of AD. For instance, women exhibit higher tau burden, and female mice show heightened microglial pathology [85, 86]. In our study, seven rhesus monkeys (5 males, 2 females; Table S1, Supporting Information) were treated with NeuroD1 AAV‐based gene therapy. However, we observed no significant sex differences in tau burden/accumulation or microglial pathology following NeuroD1 overexpression, possibly due to the limited sample size, which may have insufficient statistical power to detect potential sex‐specific treatment effects. Interestingly, female monkeys seemed to exhibit slightly poorer performance on cognitive behavioral tests compared to age‐ and treatment‐matched males. We speculate that this may be related to higher stress and anxiety levels during behavioral tests in females, though further investigation is required to confirm this hypothesis.

Age is the primary risk factor for the onset of neurodegenerative diseases such as AD. A critical question arises as to whether age modulates the efficacy of NeuroD1 AAV‐based gene therapy in AD‐like monkeys, given its relevance to translational research and clinical applicability. Due to the limited number of monkeys in each experimental group, we did not observe statistically significant age‐related differences in treatment outcomes. However, we noted several suggestive trends. For instance, older monkeys seemed to exhibit more pronounced anti‐inflammatory effects and greater recovery of glucose metabolism following gene therapy. In contrast, younger monkeys seemed to show more evident improvements in BBB integrity and cognitive functions. The molecular and cellular mechanisms underlying these potential age‐dependent therapeutic profiles warrant further investigation.

Neuroinflammation, a key driver of AD pathogenesis and progression, contributes to neuronal death and dysfunction. This process involves complex mechanisms, including the activation of NF‐κB, the NLRP3/caspase‐1 axis, TREM2, and cGAS‐STING pathways. Chronic neuroinflammation leads to glial cell dysfunction, causing them to lose homeostasis and adopt a proinflammatory phenotype that exacerbates neuronal damage. Targeting neuroinflammation has emerged as a promising therapeutic strategy for managing AD and potentially slowing its progression [87, 88]. NeuroD1 AAV‐based gene therapy mitigated excessive astrocyte and microglia activation, and reduced brain‐infiltrating leukocytes in AD‐like monkeys, suggesting its potential to alleviate neuroinflammation and slow AD progression. So far, our preliminary results from bulk RNA sequencing, single‐cell RNA sequencing, bioinformatics analysis, super‐resolution subcellular imaging, and biochemical assays indicate that: 1. regulation of neuroinflammation‐related gene expression in astrocytes, 2. modulation of the JAK1/STAT3/SOCS3 signaling pathway in astrocytes and microglia, 3. mitochondrial‐derived damage‐associated molecular pattern molecules (DAMPs) and downstream Toll‐like receptors (TLRs), and 4. Structural and functional modulation of astrocyte end‐feet and the BBB, may all contribute to the suppression of pathological neuroinflammation in AD‐like monkeys by NeuroD1 AAV‐based gene therapy.

Converting astrocytes into neurons may seem to threaten BBB integrity, yet NeuroD1 AAV‐based gene therapy aids BBB repair, despite the potential loss of astrocyte support. However, we haven't seen a significant reduction in astrocyte population in NeuroD1‐converted regions in mouse, rat, or monkey brains, suggesting that astrocytes, being proliferative, can repopulate. In fact, we observed more proliferative astrocytes (Ki67^+^) in these areas, indicating they can replenish after some are converted to neurons [15, 89, 90, 91]. Moreover, reduced reactive astrogliosis and healthier astrocytes induced by NeuroD1 AAV treatment contribute to BBB repair by stabilizing tight junctions, supporting endothelial cells, and secreting anti‐inflammatory molecules. They also support neurovascular coupling, maintain tissue homeostasis, and release growth factors like VEGF and GDNF to support endothelial cell regeneration and BBB restoration [92, 93, 94, 95, 96]. Overall, fostering astrocyte health creates a supportive environment for BBB restoration, improving its function and integrity.

The blood‐brain barrier plays a crucial role in maintaining a stable brain environment, and its damage or disruption is widely associated with neurodegenerative diseases like AD. Preserving BBB integrity is vital for the prevention and treatment of AD. Additionally, the glymphatic system, composed of astrocytes, perivascular spaces, CSF, and transport proteins, clears metabolic waste (Aβ, tau, …) and excess fluid through CSF circulation. Recently, the link between glymphatic function and neurodegenerative diseases like AD has garnered growing attention [97, 98]. NeuroD1 AAV‐based gene therapy has shown promise in restoring vascular integrity and preserving BBB function in AD‐like monkeys. After NeuroD1 treatment, we observed a reduction in tau and phospho‐tau levels, along with improved Aβ42 levels, in the CSF of NHP AD models, indicating restored glymphatic functions in the monkey brains. Thus far, our preliminary results provide some clues for subsequent research. Western blot and immunostaining of AQP4 and connexins in endothelial cell reporter mice showed that NeuroD1 overexpression partially restored the polarized distribution of AQP4 and connexins in the astrocytes of AD mice, suggesting potential molecular and cellular mechanisms for BBB repair. Moreover, dynamic tracking of the CSF tracer OA‐647 indicated that NeuroD1 overexpression alleviated glymphatic system damage in AD mice. Immunohistochemistry, western blot, and single‐cell RNA sequencing also revealed that MMP9 and PDGFβ expression and secretion may be involved in glymphatic system reconstruction via NeuroD1 overexpression. Furthermore, studies by peer researchers and our preliminary data suggest that NeuroD1 might modulate endothelial cell function by influencing pathways related to glycocalyx integrity (e.g., mucin‐type O‐glycosylation enzymes like C1GALT1/B3GNT3), though direct evidence remains limited [99].

Glia‐to‐neuron conversion, particularly through ectopic expression of transcription factors like NeuroD1, offers a promising approach for regenerative medicine for neurodegenerative disorders and CNS traumas. This strategy leverages the abundance of glial cells, astrocytes, microglia, and oligodendrocytes, in the brain to replenish lost neurons, bypassing challenges of stem cell transplantation, like immune rejection and ethical concerns. However, significant scientific discourse persists regarding the process's authenticity, reproducibility, and clinical viability [19, 20, 21, 22, 23, 24].

Pioneering studies demonstrated that NeuroD1 could directly reprogram reactive astrocytes into functional neurons in vivo, improving motor function in ischemic stroke mice and alleviating behavioral deficits in Huntington's disease mice. NeuroD1's ability to rearrange epigenetic landscapes and activate neuronal programs while suppressing glial identity drives these cell‐fate conversions. Despite these advances, critics argue that some apparent conversions may result from leaky NeuroD1 expression in preexisting neurons, rather than true reprogramming of glia. Advanced AAV tools have failed to confirm NeuroD1‐induced conversions in lineage‐traced models, suggesting overestimation in earlier reports. In stab injury models, NeuroD1‐expressing astrocytes transitioned through transit‐amplifying OLIG2^+^ intermediates, but produced immature neurons lacking full electrophysiological maturity, challenging claims of functional integration. Additionally, NeuroD1 induces apoptosis in microglia, further complicating its broad application. Conversion efficiency is influenced by factors like NeuroD1 dosage, glial reactivity, and injury timing, with optimized expression yielding up to 77% conversion in early post‐injury phases. However, optimal protocols for viral vectors, co‐factors, and lineage‐tracing techniques remain undefined [19, 20, 21, 22, 23, 24, 25, 38, 100].

In this context, this study on NeuroD1 AAV‐based gene therapy for an AD‐like NHP model fully acknowledges the multifactorial and complex nature of its therapeutic outcomes. Specifically, the observed benefits likely result from the combined effects of multiple mechanisms, including neuroregeneration, neuroprotection, reduced neuroinflammation, BBB restoration, Aβ clearance, and microenvironmental modulation. The relative contribution and intricate interplay of these mechanisms remain to be fully elucidated.

Ongoing global research using multi‐omics analyses, lineage‐tracing animals, and real‐time imaging aims to clarify these glia‐to‐neuron conversion processes, with controversies underscoring the need for standardized models. These concerted efforts promise to advance glial reprogramming technologies and revolutionize treatments for neurodegenerative diseases like AD and CNS injuries.

## 4 Conclusion

Our findings demonstrate that NeuroD1 AAV‐based gene therapy prevents neuronal loss, halts hippocampal atrophy, alleviates neuroinflammation, repairs vascular/BBB damage, restores CSF AD biomarker levels, improves glucose metabolism and enhances spatial memory in NHP AD models, thereby highlighting its therapeutic potential.

## 5 Experimental Section/Methods

### 5.1 Reagents and Antibodies

This study utilized Zoletil 50 (Virbac, France), Dexdomitor (dexmedetomidine, Orion Pharma, Finland), Propofol (Guangdong Jiabo Pharmaceutical, China), Atropine (Shanghai Quanyu Biotechnology, China), and Lidocaine (Shandong Hualu Pharmaceutical, China) as anesthetics, analgesics, and sedatives.

The following primary antibodies were utilized in this study: guinea pig anti‐GFP (ASIS BIOFARM, OBPGP003, 1:1000), chicken anti‐GFP (Abcam, ab13970, 1:1000), rabbit anti‐Tau (Dako, A0024, 1:1000), mouse anti‐pTau (S202/T205, Invitrogen, MN1020B, 1:500), rabbit anti‐pTau (T231, Abcam, ab15155, 1:1000), guinea pig anti‐NeuN (Millipore, ABN90, 1:1000), rat anti‐GFAP (Invitrogen, 130300, 1:1000), rabbit anti‐Iba1 (Fujifilm, 01919741, 1:1000), rat anti‐CD45 (BD Pharmingen, 559864, 1:500), rabbit anti‐laminin (Sigma, L9393, 1:1000), rabbit anti‐AQP4 (Proteintech, 164731AP, 1:500), mouse anti‐PECAM‐1 (Thermo Fisher, MA513188, 1:1000), and goat anti‐CD31 (Bio‐Techne, AF3628, 1:1000).

The antibodies were validated by the corresponding manufacturers:

 guinea pig anti‐GFP (ASIS BIOFARM, OBPGP003)

https://www.oasisbiofarm.net/#/productdetails?proId=a8fdb262bda2448ebba460f6f2d5e3be&exp2=%E6%8A%97%E4%BD%93

 chicken anti‐GFP (Abcam, ab13970)

https://www.abcam.cn/products/primary‐antibodies/gfp‐antibody‐ab13970.html

 rabbit anti‐Tau (Dako, A0024)

https://www.agilent.com.cn/cs/library/msds/SDS345_Chinese.pdf

 mouse anti‐pTau (S202/T205, Invitrogen, MN1020B)

https://www.thermofisher.cn/cn/zh/antibody/product/Phospho‐Tau‐Ser202‐Thr205‐Antibody‐clone‐AT8‐Monoclonal/MN1020B

 rabbit anti‐pTau (T231, Abcam, ab15155)

https://www.abcam.cn/products/primary‐antibodies/tau‐phospho‐t231‐antibody‐epr2488‐ab151559.html

 guinea pig anti‐NeuN (Millipore, ABN90)

https://www.sigmaaldrich.cn/CN/zh/product/mm/abn90

 rat anti‐GFAP (Invitrogen, 130300)

https://www.thermofisher.cn/cn/zh/antibody/product/GFAP‐Antibody‐clone‐2‐2B10‐Monoclonal/13‐0300

 rabbit anti‐Iba1 (Fujifilm, 01919741)

https://www.chem17.com/st527443/product_38693776.html

 rat anti‐CD45 (BD Pharmingen, 559864)

https://www.bdbiosciences.com/zh‐cn/products/reagents/flow‐cytometry‐reagents/research‐reagents/single‐color‐antibodies‐ruo/apc‐rat‐anti‐mouse‐cd45.559864?tab=product_details

 rabbit anti‐laminin (Sigma, L9393)

https://www.sigmaaldrich.cn/CN/zh/product/sigma/l9393

 rabbit anti‐AQP4 (Proteintech, 164731AP)

https://www.ptgcn.com/products/AQP4‐Antibody‐16473‐1‐AP.htm

 mouse anti‐PECAM‐1 (Thermo Fisher, MA513188)

https://www.thermofisher.cn/search/results?query=MA513188&focusarea=%E6%90%9C%E7%B4%A2%E5%85%A8%E9%83%A8%E6%88%96%E4%B8%8B%E5%88%97%E6%8C%87%E5%AE%9A%E5%88%86%E7%B1%BB

 goat anti‐CD31 (Bio‐Techne, AF3628)

https://www.bio‐techne.com/p/antibodies/human‐mouse‐rat‐cd31‐pecam‐1‐antibody_af3628.

DAPI and Neuro Trace Nissl were obtained from Roche (10236276001, 1:1000) and Thermo Fisher Scientific (N21482 1:100), respectively.

### 5.2 AAV Preparation

In this study, recombinant adeno‐associated viruses (AAVs) were used as viral vectors. The AAVs employed to generate AD‐like monkeys were prepared according to a previously described protocol [18]. For the NeuroD1 gene therapy, AAV9 GFAP(CMVe)::NeuroD1 and AAV9 GFAP::GFP vectors were generated by packaging the NeuroD1 gene and GFP reporter into recombinant AAVs. This allowed for the expression of both NeuroD1 and GFP under the GFAP promoter, specifically targeting astrocytes. In contrast, the control vectors (AAV9 GFAP(CMVe)::inverted NeuroD1 and AAV9 GFAP::GFP) expressed only GFP, since the control vector AAV9 GFAP(CMVe)::inverted NeuroD1 does not produce a functional protein. All AAVs utilized in this study were packaged and manufactured by PackGene Biotech Inc. (Guangzhou, China). Briefly, the AAV production process included bacterial transformation, plasmid preparation, HEK293 cell co‐transfection, crude purification, concentration and ultracentrifugation, sterilization, and rigorous quality control tests.

### 5.3 Animals

Seventeen adult rhesus macaques, comprising 2 females and 15 males, ranging in age from 5 to 15 years, were used in this study (Table S1, Supporting Information). The NHP studies were conducted in compliance with the standards established in the eighth edition of the Guide for the Care and Use of Laboratory Animals (NRC, 2011) and recommendations outlined in the Weatherall report (https://acmedsci.ac.uk/policy/policy‐projects/use‐of‐non‐human‐primates‐in‐research).

The macaques were housed in easily sanitized cages located in climate‐controlled rooms at Guangdong Yuan Xi Biotech Co., Ltd. (Guangzhou, China), which holds full accreditation from the Association for Assessment and Accreditation of Laboratory Animal Care (AAALAC) International. All macaques were housed in a compliant environment, as suggested by the Weatherall report. Their cages were constructed with a minimum height of 1.8 m and a floor area exceeding 3 m^2^, surpassing the requirements set forth by Directive 2010/63/EU. These cages provided shade and shelter, ensuring protection from unfavorable environmental conditions. The ambient humidity was maintained between 40% and 70%, with a 12‐h light/12‐h dark cycle, and background noise kept to a minimum. Food and water were provided regularly. A team of four staff members, including technicians and veterinarians, were responsible for their daily care and management.

All experimental procedures had been approved by the Institutional Animal Care and Use Committee (IACUC) of Guangdong Yuan Xi Biotech Co., Ltd. (IACUC No. YXSW‐2021–002) and Jinan University (IACUC No. 20210806‐03). The experimental procedures also strictly adhered to the “Guide for the Care and Use of Laboratory Animals” and “The Use of Nonhuman Primates in Research,” both established by the Institute of Laboratory Animal Science in 2006, ensuring both personnel safety and animal welfare. The monkeys were randomly assigned into either the control or NeuroD1 treatment groups.

### 5.4 Generation of the NHP Model with AD‐Like Pathology

The technical details regarding the generation of the NHP model with AD‐like pathology, which was utilized in this study to assess the efficacy of NeuroD1 AAV‐based gene therapy, are not included in this manuscript due to space constraints. For a comprehensive description of the methodologies employed, please refer to the Methods section of our prior publication [18]. We apologize for any inconvenience.

### 5.5 Surgical Procedures and Stereotactic Injection of AAVs

Monkeys were fasted for 12 h prior to surgery to avoid aspiration of food into their trachea in case of vomiting under general anesthesia during surgery. Atropine (0.02 mg/kg) was administered intramuscularly before surgery to inhibit respiratory secretions. Anesthesia induction was then carried out using Zoletil 50 (5–10 mg/kg, intramuscular) combined with Dexdomitor (10–20 mcg/kg, intramuscular). To alleviate pain, lidocaine (1–4 mg/kg, subcutaneous or intramuscular) was administered as a local anesthetic before surgery. During surgery, anesthesia was maintained with Propofol (2.5–3.5 mg/kg, intravenous).

After anesthesia, the monkeys were connected to veterinary vital signs monitor (JRTYL, Hunan, China) for continuous monitoring of their stable vital signs. Throughout the procedure, the monkeys' vital signs were diligently monitored, including blood oxygen levels (>95%), heart rate (150–220 bpm), respiratory rate (10–25 breaths/min), and blood pressure (mean >60 mmHg, systolic >90 mmHg). Subsequently, the monkey's head was secured in a stereotaxic frame (RWD Life Science, Shenzhen, China), and the scalp was thoroughly disinfected and cleaned. The precise stereotaxic injection sites were determined based on the T1‐weighted MRI image data of the monkey's brain. More precisely, 6 sets of coordinates for stereotaxic injection were meticulously determined for each monkey, ensuring that the injection sites were evenly distributed throughout the hippocampus. This strategic approach aimed to optimally cover all hippocampal sub‐regions, including the dentate gyrus (DG), CA1, CA2, CA3, CA4, subiculum, approximately the upper one‐third of the parahippocampal gyrus, and the anterior half of the retrosplenial cortex. Once the target sites were identified, a hand‐held cranial drill (RWD Life Science, Shenzhen, China) was used to create small holes into the skull. A microinjection pump (World Precision Instruments, USA) and a microsyringe (Hamilton Company, USA) were then utilized to inject AAVs (7.5 µL per site) at each location. The injection rate was precisely controlled using a Micro4 controller (10^11–12^ GC/mL, flow rate: 800 nL/min). Following each injection, the site was left undisturbed for 10 min before the syringe was slowly removed. Lastly, the wound and surrounding skin were meticulously cleaned, and penicillin sodium (100,000 U/kg, intramuscular) was administered for three consecutive days post‐surgery to prevent infection.

### 5.6 Immunohistochemistry (IHC)

The macaques were anesthetized and subsequently euthanized with an overdose of pentobarbital sodium (100 mg/kg). Immediately following, a syringe was inserted into the macaque's heart. A cold (4 °C) saline solution containing heparin was injected to clear the blood vessels, followed by the infusion of 4% formaldehyde (PFA) solution. The brain was then dissected post‐perfusion. The entire brain was immersed in 4% PFA for 12 h. Subsequently, the brain was sectioned into 1 cm coronal sections using a monkey brain matrix (Shanghai Tow Intelligent Technology, China). These sections were further fixed and dehydrated in 4% PFA (24 h) and gradient sucrose solutions (10%, 20%, 30%). The dehydrated brain sections were then embedded in Tissue‐Tek O.C.T. Compound (Sakura Finetek, USA) and serially sectioned at 50 mm thickness in the coronal plane with a cryostat (CryoStar NX50, Thermo Fisher Scientific, USA).

The brain sections were washed in PBS for 3 times, then incubated with a blocking solution consisting of 5% donkey serum, 3% bovine serum, and 0.5% Triton for 1 h. After blocking, the slices were incubated with the primary antibodies, prepared in blocking solution, at 4 °C for 24 h. Following primary antibody incubation, the slices were washed 3 times with PBS. A mixture of secondary antibody and DAPI was then applied, and the slices were incubated for 2 h. The secondary antibodies used were Alexa Fluor 488, Alexa Fluor 555, or Alexa Fluor 647. After incubation, the slices were washed in PBS for 3 more times. Finally, the slices were mounted with VECTASHIELD anti‐fading mounting medium (VECTOR Laboratories, USA) and sealed with nail polish.

### 5.7 Wide‐Field and Confocal Fluorescence Imaging and Quantification of IHC Results

Most fluorescence images were captured using modular microscope user interface software (ZEN 2.5, 64‐bit version, Carl Zeiss, Jena, Germany) on either a conventional wide‐field microscope (Carl Zeiss Axio Imager Z2, Jena, Germany) or a confocal microscope (Carl Zeiss LSM 880 with Airyscan, Jena, Germany). For the brain sections that were directly compared, all IHC procedures were conducted concurrently, and identical image acquisition settings were used.

For the direct comparison of hippocampal sections, all IHC procedures were carried out concurrently, and the microscope image acquisition settings were kept consistent. To determine the density of cells with specific markers, we calculated the 2‐dimensional (2D) cell density (cells per mm^2^) by dividing the number of cells in a region of interest (ROI) by the total area of the ROI, and the 3‐dimensional (3D) cell density (cells per mm^3^) by multiplying the 2D density by the number of brain slices required to form a 1 mm thick stack. To ensure systematic random sampling for a particular IHC marker, we selected 9 (3 × 3) or 16 (4 × 4) evenly and symmetrically distributed ROIs (each containing at least 100+ specific cells) in a hippocampal region of a brain slice, and 5–10 evenly spaced brain slices (at intervals of 400–2000 µm, depending on the size of the hippocampal region) from each animal. Finally, *N* in this study represents the number of individual animals used.

### 5.8 Nissl Staining

The Nissl staining reagent used in this study was NeuroTrace NISSL 530/615 Red Fluorescent Nissl Stain (Thermo Fisher Scientific, USA). This staining method was highly selective for neuron‐specific Nissl substances and demonstrated greater sensitivity compared to traditional histological dyes like toluidine blue or cresol violet. The brain slices for Nissl staining were 50 mm thick and were immersed in a solution of sugar and PFA. Following this, the treated slices were washed three times with PBS. The Nissl stain was then diluted in PBST solution at a 1:100 ratio. An appropriate amount of the diluted stain was applied to the brain slices, which were then incubated at room temperature for 30 min. Subsequently, the slices were washed with PBS for 3 times and mounted with a sealing agent.

### 5.9 Magnetic Resonance Imaging (MRI) and Positron Emission Tomography (PET)

Before each MRI and PET scan, the macaques were fasted for a minimum of 6 h and anesthetized using an intramuscular injection of Zoletil 50 and Dexdomitor.

All MRI scans were conducted using a 3.0‐T MRI scanner (Discovery MR750 3.0T, GE Healthcare, USA) that was equipped with an 8‐channel customized head coil designed for macaques (Medcoil MK80, Suzhou, China) at the PET/CT‐MRI center located in the First Affiliated Hospital of Jinan University. MRI data were collected with GE Healthcare Advantage Workstation (version AW Server 3.2). The whole‐brain images were obtained using 3 different sequences: a 3D Bravo T1 sequence with a repetition time (TR) of 8.4 ms, echo time (TE) of 3.5 ms, slice thickness of 0.5 mm, matrix size of 300 × 300, and field of view (FOV) of 15 × 15 cm; a CUBE T2 sequence with a TR of 2500.0 ms, TE of 108.9 ms, slice thickness of 0.5 mm, matrix size of 320 × 320, and FOV of 15 × 15 cm; and a T2 FLAIR sequence with a TR of 8400.0 ms, TE of 151.7 ms, slice thickness of 1.0 mm, matrix size of 256 × 256, and FOV of 16 × 16 cm.

The ^18^F‐FDG PET scans were performed with a 128‐slice time‐of‐flight PET/CT scanner (GE Discovery PET/CT 690 Elite, GE Healthcare, USA) at the PET/CT‐MRI center in the First Affiliated Hospital of Jinan University. The radiochemical purity of the ^18^F‐FDG exceeded 99%. While awake, each monkey received an intravenous injection of ^18^F‐FDG (∼150 MBq, ranging from 0.3 to 0.5 mCi/kg) via the posterior saphenous vein. Fifty minutes later, the monkey was positioned inside the scanner. Its head was secured with a stereotactic frame, and a static PET scan was conducted. The procedure involved an initial CT scan, succeeded by a 10‐min static PET data acquisition at 60 min post‐injection. The PET data underwent attenuation correction through integrated CTAC technology. Subsequently, the PET/CT and MR images were co‐registered and analyzed using PMOD software (version 4.2). The standard uptake value (SUV) for each region of interest (ROI) was quantitatively extracted, with decay correction applied back to the time of radioligand injection, based on an individualized atlas.

### 5.10 Methodology and Software for MRI 3D Reconstruction, Brain Region Segmentation, and Hippocampal Volume Calculation

MRI 3D reconstruction and hippocampal volume calculation were performed using Brainsight software (Rogue Research, Montréal, Québec, Canada). In monkey coronal MRI sections, the boundaries of the hippocampus were delineated across 18 consecutive 1 mm‐thick sections, spanning from +16 mm rostral to EBZ (ear bar zero) to –1 mm caudal to EBZ. A 3D reconstruction of the hippocampus was automatically rendered from these contours, and the total hippocampal volume was computed. Segmentation of monkey brain regions was guided by the reference atlas *A Combined MRI and Histology Atlas of the Rhesus Monkey Brain in Stereotaxic Coordinates* (Saleem & Logothetis, 2nd ed., Academic Press). Briefly, coronal fluorescence microscopy images of individual monkey brains were matched to the closest corresponding coronal section in the atlas. The subregional boundaries of the brain, as defined in the atlas, were then superimposed onto the fluorescence microscopy images to achieve precise region‐of‐interest segmentation.

### 5.11 Single Molecule Array (Simoa)‐Based CSF Analysis

This study employed Simoa technology to identify key AD markers in the cerebrospinal fluid (CSF) of rhesus monkeys. The CSF was collected using a 22 G puncture needle, targeting between L4–L5 of the monkeys' spines. Immediately after collection, the CSF samples were processed in a high‐speed centrifuge, which spun at 10,000 *g* at 4 °C for 5 min. The resulting supernatant was then frozen at −80 °C for subsequent analysis. The AD markers analyzed included total Tau, phospho‐Tau 181, phospho‐Tau 231, Aβ42, Aβ40, and NFL. These markers were detected using Quanterix ultrasensitive digital biomarker detection technology and the Simoa HD‐X Analyzer (Quanterix, Billerica, MA, USA). In this study, the following Simoa kits were used: the NF‐light V2 Advantage kit (104073), the Neurology 3‐Plex A Kit (101995), the pTau‐181 V2 Advantage Kit (103714), and the pTau‐231 Advantage Kit (102292, Quanterix, USA).

### 5.12 Animal Behavioral Studies

The Wisconsin General Test Apparatus (WGTA) was used to perform the “delayed response” (DR) task. In this task, a monkey sat in its home cage facing a tray with three food wells, each covered by identical swing‐away lids. The experimenter would bait one of the wells with food in view of the monkey, cover it, and then lower an opaque screen to obscure the tray from the monkey's sight. Following a designated delay period, defined as the “memory retention interval,” the screen was pulled up, allowing the monkey to retrieve the food from the baited well based on its working memory. Over the course of 30 trials per day, food reinforcers were randomly placed in one of the three wells. The initial memory retention interval for each monkey was set at 5 s and was progressively increased using a stepwise procedure. Specifically, the interval was increased whenever the monkey successfully identified the baited well in ≥26 out of 30 trials per day for three consecutive days, indicating mastery of that particular interval. If a monkey failed to master a memory retention interval within a week, the DR task was discontinued, and the last interval successfully mastered was recorded for analysis.

In this study, the macaques underwent 3 rounds of testing both before and after human tau (hTau) overexpression, as well as following NeuroD1 AAV‐based treatment. Each testing round typically took approximately four weeks to complete for the majority of the monkeys. The entire experimental process was documented using video recording.

### 5.13 Non‐Human Primate Bulk‐RNA Sequencing

The samples for bulk‐RNA sequencing were collected from the hippocampi of the monkeys, and subsequently transported on dry ice to Shanghai Biotechnology Company (Shanghai, China) for further processing. Briefly, total RNA was extracted using the TransZol Up Plus RNA Kit (code #ER501‐01‐V2). The RNA integrity was assessed and a RNA integrity number (RIN) was determined for each sample with an Agilent 2100 bioanalyzer (Agilent technologies, Santa Clara, CA, USA). The qualified total RNA was further purified using the RNAClean XP Kit (Beckman Coulter, Inc., Kraemer Boulevard Brea, CA, USA) in conjunction with the RNase‐Free DNase Set (Cat#79254, QIAGEN GmbH, Germany).

The total RNA was purified and subsequently segmented, undergoing first‐strand cDNA synthesis, second‐strand cDNA synthesis, adapter ligation, rRNA depletion, amplification, and additional steps, followed by the construction of the sequencing sample library. These procedures were executed utilizing the SMARTer Stranded Total RNA‐Seq Kit v2 (Clontech, Mountain View, CA, USA), VAHTS DNA Clean Beads (#N411, Vazyme, Nanjing, Jiangsu, China), and the ExKubit dsDNA HS test kit (ExCell Biotech Co., Ltd., Shanghai, China). Subsequently, the library underwent purification with the Qubit 2.0 Fluorometer (Life Technologies, Carlsbad, CA, USA) for quantitative analysis and verification using the Agilent 2100 bioanalyzer (Agilent Technologies, Santa Clara, CA, USA). The library was diluted to 10 pM with the Illumina cBot system to generate clusters, followed by sequencing on the Illumina NovaSeq 6000 Sequencing System (Illumina, San Diego, CA, USA). The sequencing and library construction were carried out by Shanghai Biotechnology Company.

### 5.14 Gene Expression Data Analysis

Raw sequencing reads underwent preprocessing to eliminate rRNA reads, sequencing adapters, short fragments, and other substandard reads. We employed HISAT2 (version 2.0.4) to align the cleaned reads to the Genome Reference Consortium Human Build 38 (GRCh38) reference genome, allowing for a maximum of two mismatches. Subsequently, StringTie (version 1.3.0) was executed with reference annotations to generate Fragments Per Kilobase per Million (FPKM) values for established gene models. Differentially expressed genes (DEGs) were identified using StringTie, with significance determined by the false discovery rate (FDR) in multiple tests. Fold‐changes were calculated based on FPKM values across samples. DEGs were selected using the following filter criteria: FDR ≤ 0.05 and fold‐change ≥ 2.

### 5.15 Statistics

Image processing was conducted utilizing ZEN 2.5 (Carl Zeiss, Germany), ImageJ (version 1.52, 64‐bit, National Institutes of Health, USA) and RadiAnt DICOM Viewer (version 2021.2, 64‐bit). The tissue volume measurements and 3D reconstructions were obtained with Brainsight software (Rogue Research, Montréal, Québec, Canada). Statistical analyses were carried out using GraphPad Prism v8.4.2 (GraphPad Software, La Jolla, CA, USA), with datasets being evaluated for normality before significance tests. For comparisons between two groups, a two‐tailed Student's *t*‐test was employed. When comparing among multiple groups, a one‐way ANOVA with Tukey's post hoc test was utilized to determine significance. Longitudinal data collected at various time points was analyzed using repeated measures ANOVA with Tukey's post hoc test. Significance was defined as a *p*‐value < 0.05. Results are presented as mean ± SEM, and data collection and analysis were performed blindly whenever feasible.

## Author Contributions

W.L., G.C., and Z.W. supervised the entire project. Z.J. and Y.Q. performed the surgical procedures and stereotactic injections, conducted the immunostaining assays, and completed the wide‐field and confocal imaging analyses; H.W., J.W., B.L., F.B. carried out the MRI and PET scans; B.L. and F.B. performed the animal behavioral studies; Z.J., B.L., and F.B. performed the Simoa‐based CSF analysis; Z.J., Y.Q., and S.L. prepared the samples for bulk‐RNA sequencing; Z.J., Y.Q., and W.L. conducted the statistical analysis; W.L. and G.C. wrote the manuscript. All authors approved the final version of the paper.

## Conflicts of Interest

Gong Chen is a cofounder of NeuExcell Therapeutics Inc. The other authors have no conflicts of interest to declare.

## Supporting information




**Supporting File**: advs74707‐sup‐0001‐SuppMat.pdf.

**Supporting Movie**: advs74707‐sup‐0002‐ Movie S1.avi.

**Supporting Data**: advs74707‐sup‐0003‐DataFile.xlsx.

## Data Availability

The data that support the findings of this study are available in the article and its Supporting Information files. Bulk RNA‐seq data generated in this study have been deposited in the National Center for Biotechnology Information at https://www.ncbi.nlm.nih.gov/, accession number PRJNA1304051. Raw data are provided with this paper. The original MRI and immunostaining data of macaques that support the findings of this study are openly available in OMIX, China National Center for Bioinformation at https://www.cncb.ac.cn/, accession number PRJCA046140 (MRI) and PRJCA046807 (immunostaining). https://www.ncbi.nlm.nih.gov/
