# Supplementary material for: A NeuroD1 AAV‐Based Gene Therapy for Functional Brain Repair in Alzheimer's Disease‐Like Non‐Human Primate Model
Source: Adv Sci (Weinh). 2026 Mar 10;13(27):e20239. doi: 10.1002/advs.202520239 (PMC13170240; doi:10.1002/advs.202520239)
Supplement: Supplementary file 1 — Supporting File: advs74707‐sup‐0001‐SuppMat.pdf. [file ADVS-13-e20239-s002.pdf]

Supporting Information

A NeuroD1 AAV-based gene therapy for functional brain repair in Alzheimer's disease-like non-human primate model

Zhouquan Jiang, Yongpeng Qin, Bin Luo, Fan Bai, Jiangyue Liu, Long Ma, Shu He, Rongjie Chen, Yuchen Wang, Shanggong Liu, Ying Sun, Yi Chen, Shuo Zhang, Jiaqi Liang, Feng Liao, Huiyi Wei, Junjie Wei, Lu Wang, Hao Xu, Zheng Wu, Gong Chen*, and Wenliang Lei**

Table S1. Details of the monkeys used in this study.

Animal No.	Sex	Age (year)	Virus injection	The experiments that the animal was used for
**C01**	M	8	Control AAV	Immunostaining (hTau/pTau), Immunostaining (NeuN/GFAP/Iba1/CD45/Laminin/ PECAM1/AQP4), Nissl staining
**C02**	M	7	Control AAV	Immunostaining (hTau/pTau), Immunostaining (NeuN/GFAP/Iba1/CD45/Laminin/ PECAM1/AQP4), Nissl staining
**C03**	M	5	Control AAV	Immunostaining (hTau/pTau), Immunostaining (NeuN/GFAP/Iba1/CD45/Laminin/ PECAM1/AQP4), Nissl staining, Bulk-RNA sequencing
**C04**	M	8	Control AAV	Immunostaining (hTau/pTau), Immunostaining (NeuN/GFAP/Iba1/CD45/Laminin/ PECAM1/AQP4), Nissl staining, Bulk-RNA sequencing
**C05**	M	9	Control AAV	Immunostaining (hTau/pTau), Immunostaining (NeuN/GFAP/Iba1/CD45/Laminin/ PECAM1/AQP4), Nissl staining, Bulk-RNA sequencing
**T01**	M	9	AAV expressing hTau + AAV expressing GFP	Immunostaining (hTau/pTau), Nissl staining
**T02**	M	7	AAV expressing hTau + AAV expressing GFP	Immunostaining (GFP), Immunostaining (hTau/pTau), Immunostaining (NeuN/GFAP/ Iba1/CD45/Laminin/PECAM1/AQP4/NeuroD1), Nissl staining
**T03**	M	10	AAV expressing hTau + AAV expressing GFP	Immunostaining (GFP), Immunostaining (hTau/pTau), Immunostaining (NeuN/GFAP/ Iba1/CD45/Laminin/PECAM1/AQP4/NeuroD1), Nissl staining, MRI/PET-CT, Behavioral tests (learning, memory), Bulk-RNA sequencing
**T04**	M	10	AAV expressing hTau + AAV expressing GFP	Immunostaining (GFP), Immunostaining (hTau/pTau), Immunostaining (NeuN/GFAP/ Iba1/CD45/Laminin/PECAM1/AQP4/NeuroD1), Nissl staining, MRI/PET-CT, Behavioral tests (learning, memory), Bulk-RNA sequencing

**T05**	M	10	AAV expressing hTau + AAV expressing GFP	Immunostaining (GFP), Immunostaining (hTau/pTau), Immunostaining (NeuN/GFAP/Iba1/CD45/Laminin/PECAM1/AQP4/NeuroD1), Nissl staining, MRI/PET-CT, Behavioral tests (learning, memory), Bulk-RNA sequencing
**N01**	F	14	AAV expressing hTau + AAV expressing NeuroD1&GFP	Immunostaining (GFP), CSF collection & analysis (AD biomarkers), Immunostaining (NeuN/GFAP/Iba1/CD45/Laminin/PECAM1/AQP4/NeuroD1), Nissl staining, MRI/PET-CT, Behavioral tests (learning, memory), Bulk-RNA sequencing
**N02**	F	15	AAV expressing hTau + AAV expressing NeuroD1&GFP	Immunostaining (GFP), CSF collection & analysis (AD biomarkers), Immunostaining (NeuN/GFAP/Iba1/CD45/Laminin/PECAM1/AQP4/NeuroD1), Nissl staining, MRI/PET-CT, Behavioral tests (learning, memory), Bulk-RNA sequencing
**N03**	M	14	AAV expressing hTau + AAV expressing NeuroD1&GFP	Immunostaining (GFP), CSF collection & analysis (AD biomarkers), Immunostaining (NeuN/GFAP/Iba1/CD45/Laminin/PECAM1/AQP4/NeuroD1), Nissl staining, MRI/PET-CT, Behavioral tests (learning, memory), Bulk-RNA sequencing
**N04**	M	15	AAV expressing hTau + AAV expressing NeuroD1&GFP	Immunostaining (GFP), CSF collection & analysis (AD biomarkers), Immunostaining (NeuN/GFAP/Iba1/CD45/Laminin/PECAM1/AQP4/NeuroD1), Nissl staining, MRI/PET-CT, Behavioral tests (learning, memory), Bulk-RNA sequencing
**N05**	M	15	AAV expressing hTau + AAV expressing NeuroD1&GFP	Immunostaining (GFP), CSF collection & analysis (AD biomarkers), Immunostaining (NeuN/GFAP/Iba1/CD45/Laminin/PECAM1/AQP4/NeuroD1), Nissl staining, MRI/PET-CT, Behavioral tests (learning, memory), Bulk-RNA sequencing
**N06**	M	9	AAV expressing NeuroD1	Immunostaining (NeuroD1)
**N07**	M	8	AAV expressing hTau + AAV expressing NeuroD1&GFP	Immunostaining (GFP), Immunostaining (NeuN/GFAP/Iba1/CD45/Laminin/PECAM1/AQP4/NeuroD1), Nissl staining

This table provides an overview of the monkeys used in this study, detailing their animal No., sex, age, the viruses injected into the hippocampus, and the experiments in which they participated. The study used 17 adult *Macaca mulattas* (15 males and 2 females, age 5~15 years). Control AAV: AAV CAG::FRT-hTau; AAV expressing hTau: AAV Syn::FLPo + AAV CAG::FRT-hTau; AAV expressing GFP: AAV GFAP(CMVe)::inverted NeuroD1 + AAV GFAP::GFP; AAV expressing NeuroD1: AAV GFAP(CMVe)::NeuroD1; AAV expressing NeuroD1&GFP: AAV GFAP(CMVe)::NeuroD1 + AAV GFAP::GFP.

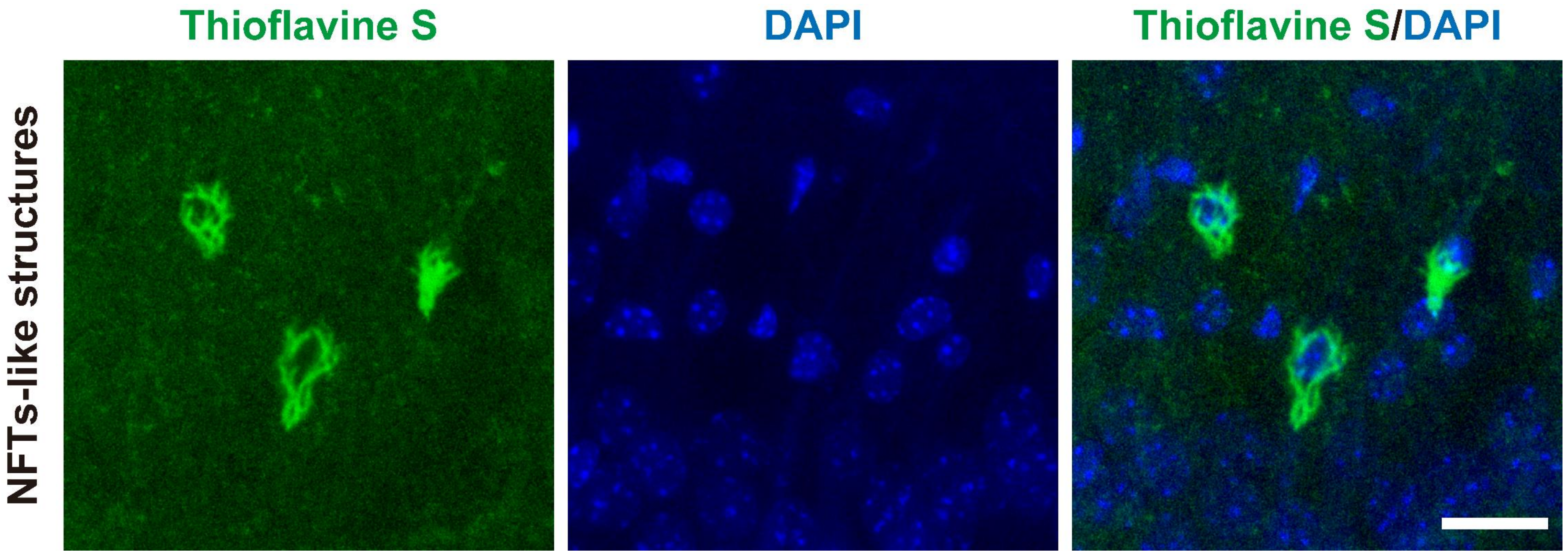

Figure S1. Thioflavine S staining reveals NFT-like aggregates within hippocampal neurons of our AD-like monkeys. Representative high-magnification confocal images of Thioflavine S-stained hippocampal neurons show intracellular thread-like structures resembling the NFTs observed in AD patients. Scale bars, 20 μm.

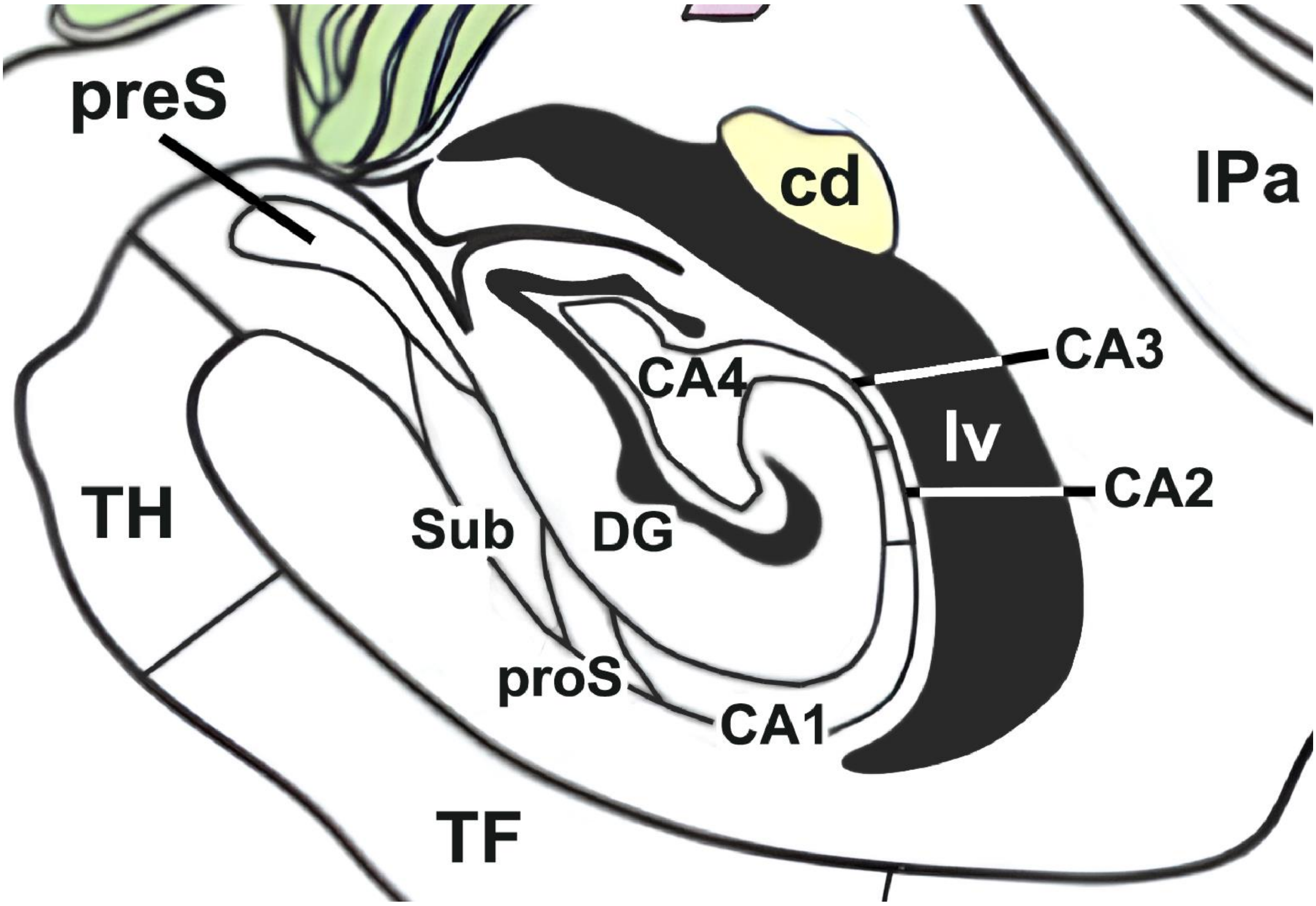

Figure S2. Hippocampal regions used for area/volume calculations in Fig. 1E, as shown in the monkey brain atlas.

The hippocampal regions analyzed for area/volume in Fig. 1E are depicted in coronal sections (+7 mm rostral to EBZ) from the "*A Combined MRI and Histology Atlas of the Rhesus Monkey Brain in Stereotaxic Coordinates*" (Saleem & Logothetis, 2nd ed.). Abbreviations: lv, lateral ventricle; CA1, CA1 subfield of hippocampus; CA2, CA2 subfield of hippocampus; CA3, CA3 subfield of hippocampus; CA4, CA4 subfield of hippocampus; DG, dentate gyrus; cd, caudate nucleus; preS, presubiculum; proS, prosubiculum; Sub, subiculum.

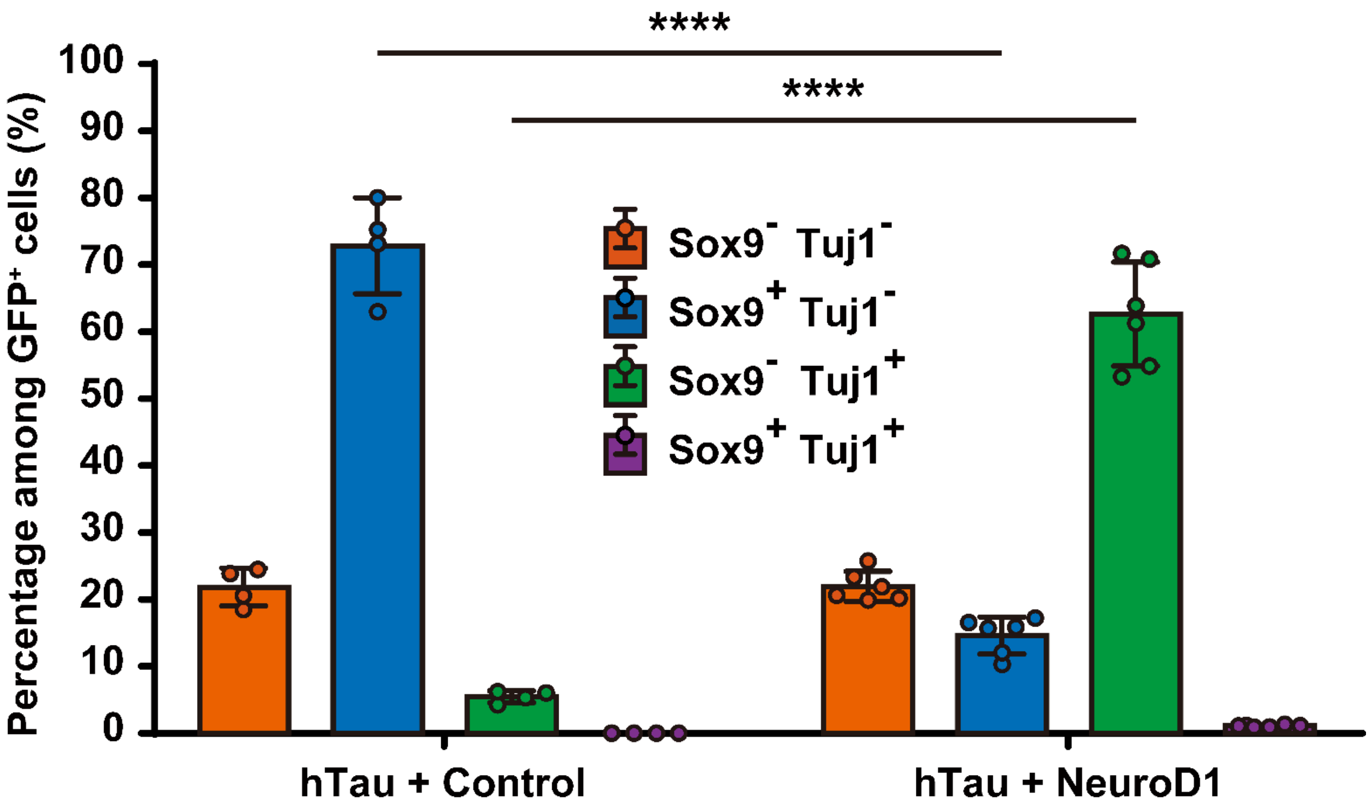

Figure S3. The shift in cellular identity following NeuroD1 overexpression in monkey hipppocampus. Quantitation of the shift in cellular identity between the control group and the NeuroD1 treatment group, as indicated by the percentage of cells co-expressing GFP with either Sox9 or Tuj1. ****$P < 0.0001$, One-way ANOVA with Tukey's post hoc test, Control group $N = 4$, NeuroD1 group $N = 6$.

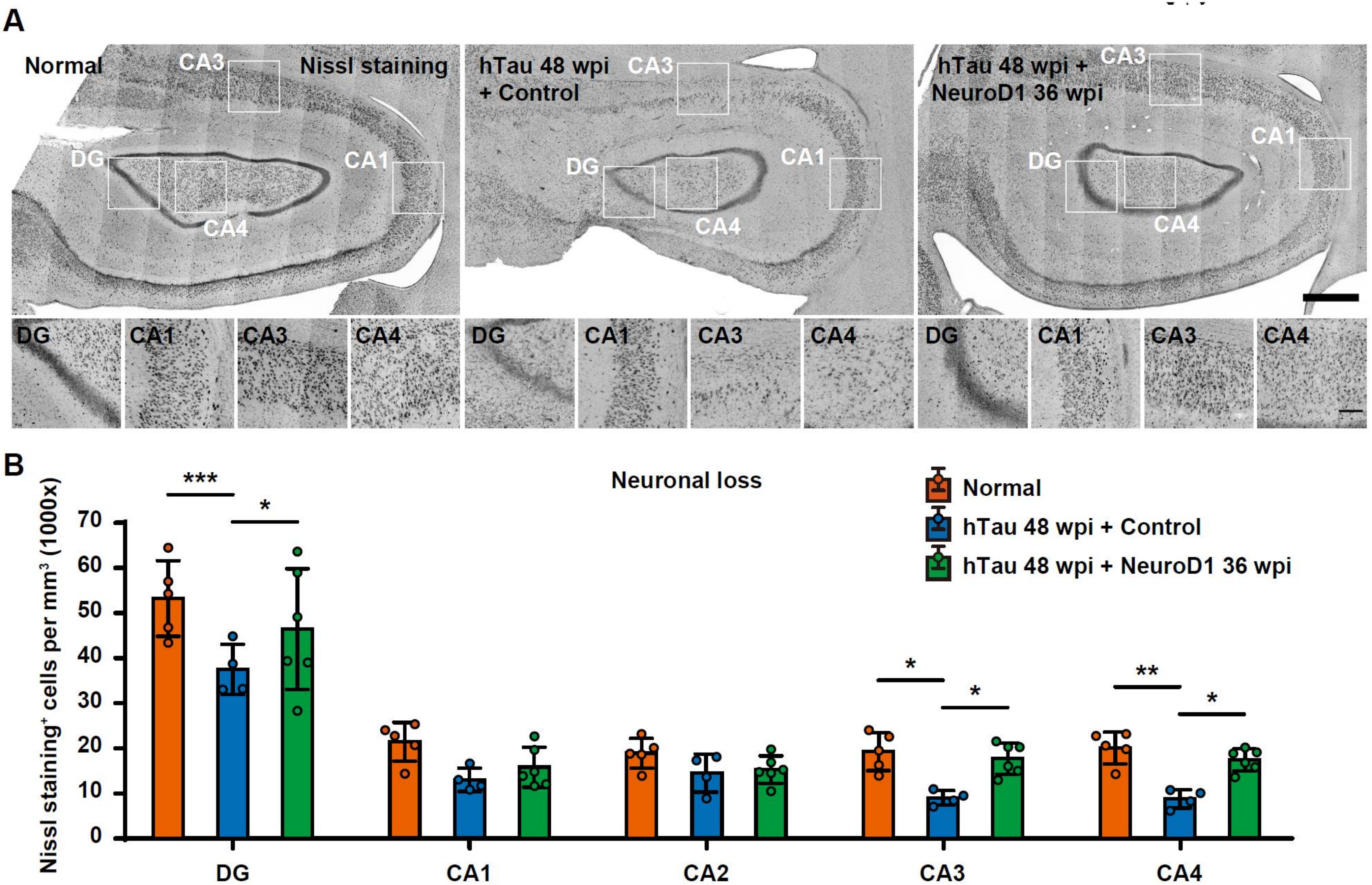
A
Normal
Nissl staining
CA3
DG
CA1
CA4
hTau 48 wpi
+ Control
hTau 48 wpi +
NeuroD1 36 wpi
DG
CA1
CA3
CA4
B
Neuronal loss
Normal
hTau 48 wpi + Control
hTau 48 wpi + NeuroD1 36 wpi
Nissl staining⁺ cells per mm³ (1000x)
0
10
20
30
40
50
60
70
***
*
**
DG
CA1
CA2
CA3
CA4

Figure S4. NeuroD1 AAV-based gene therapy prevents neuronal damage and loss in AD-like monkeys.

A) Representative images of Nissl staining reveal a significant decrease in the number of Nissl-stained cells in the control group, and a notable increase in the number of Nissl-stained cells in the NeuroD1 treatment group. Scale bars, 1 mm and 200 µm (inset).

B) Quantification of Nissl-stained cell densities to assess neuronal loss in AD-like monkeys and evaluate neuronal regeneration/protection after NeuroD1 overexpression. ***$P < 0.001$, **$P < 0.01$, *$P < 0.05$, One-way ANOVA with Tukey's post hoc test, Normal group N = 5, Control group N = 4, NeuroD1 group N = 6.

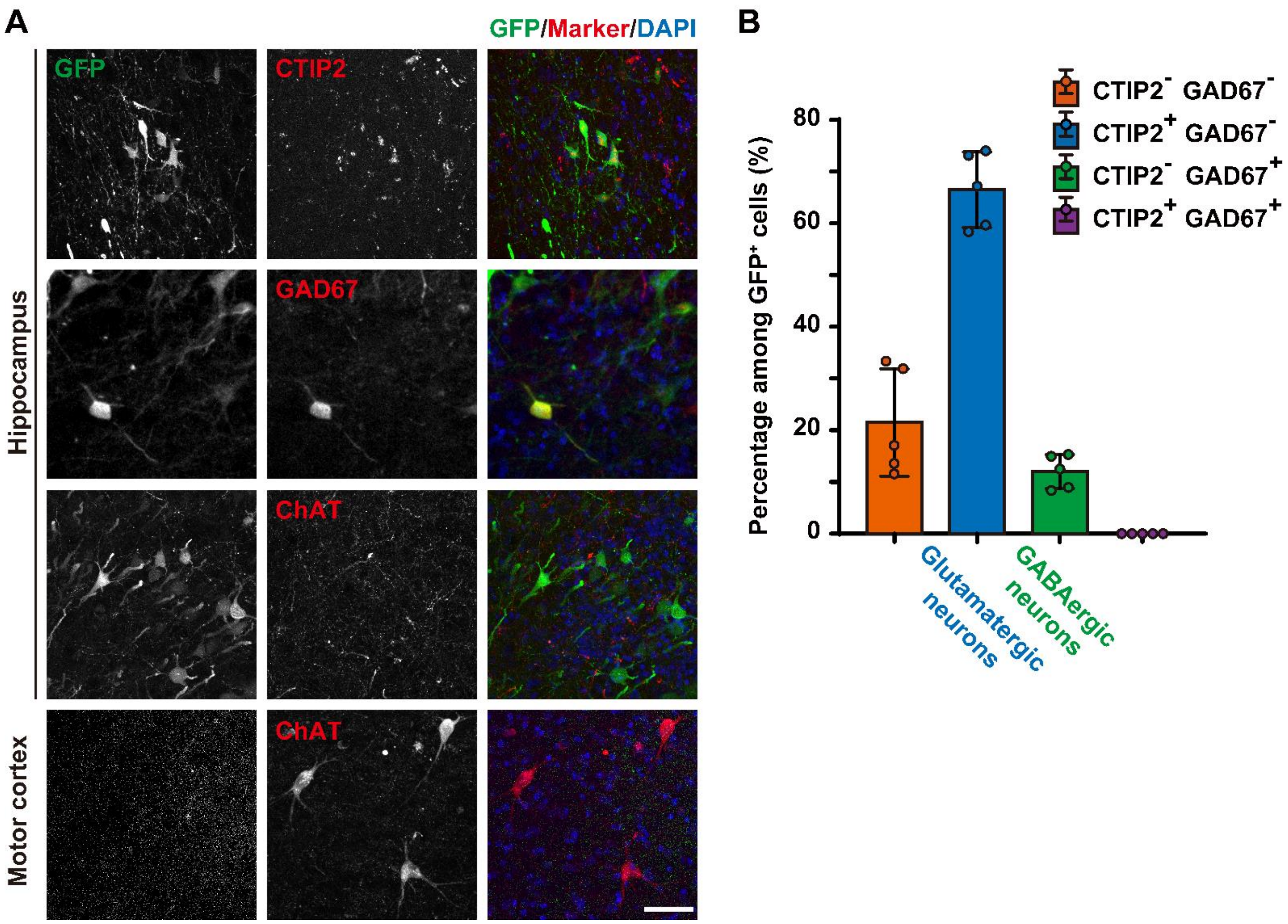

Figure S5. Neuronal subtype classification of GFP^+ neurons in AD-like monkeys following NeuroD1 AAV-based gene therapy.

A) Representative images of CTIP2, GAD67, ChAT immunostaining exhibit specific neuronal subtypes among all the GFP^+ neurons in the monkey hippocampus and motor cortex following hippocampal NeuroD1 overexpression. Scale bars, 50 μm.

B) Quantification of the percentage of $CTIP2^-$ $GAD67^-$, $CTIP2^+$ $GAD67^-$, $CTIP2^-$ $GAD67^+$, and $CTIP2^+$ $GAD67^+$ cells among GFP^+ cells after NeuroD1 overexpression. N = 5.

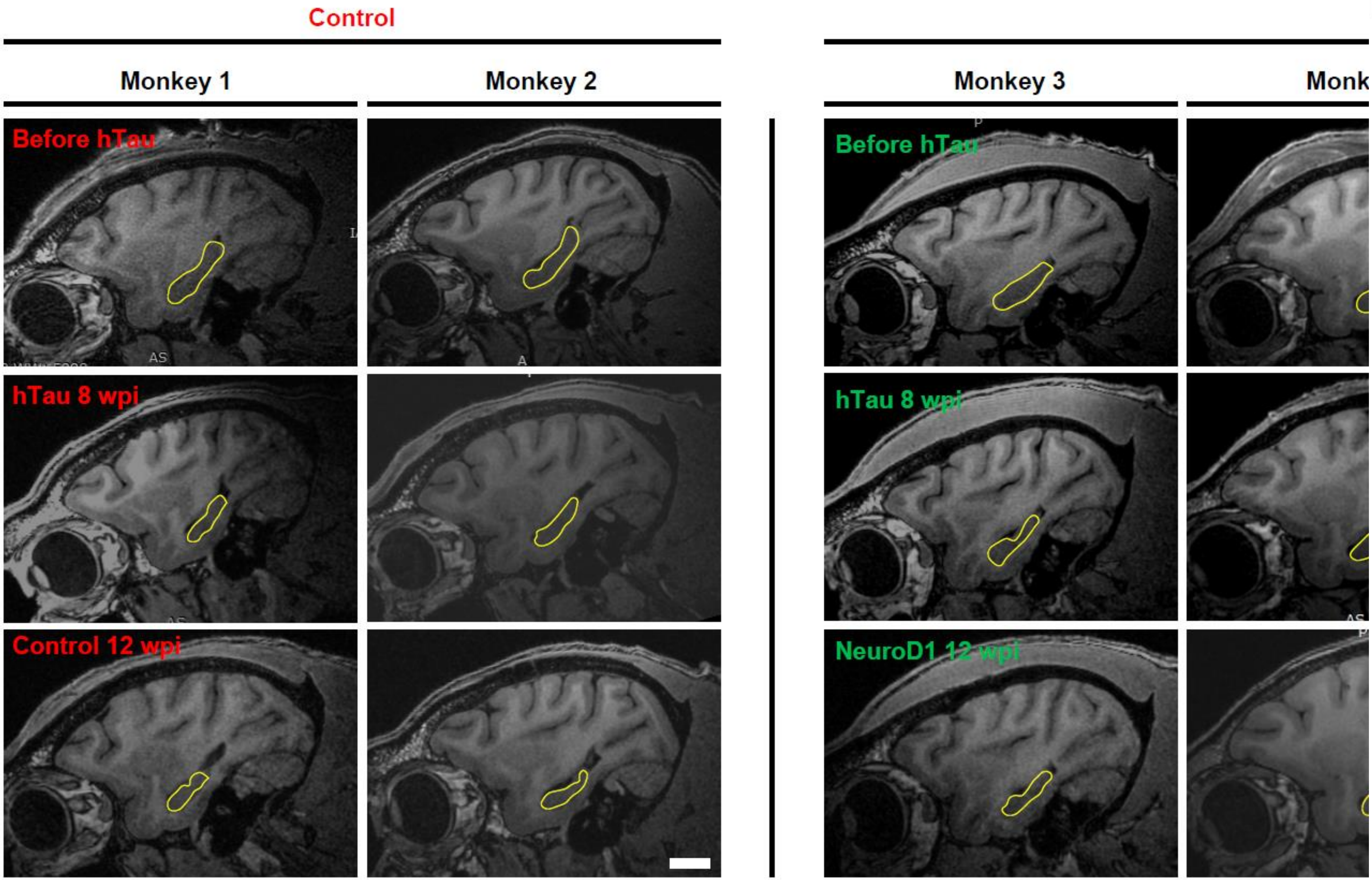
Control
Monkey 1
Monkey 2
Monkey 3
Monk
Before hTau
hTau 8 wpi
Control 12 wpi
Before hTau
hTau 8 wpi
NeuroD1 12 wpi

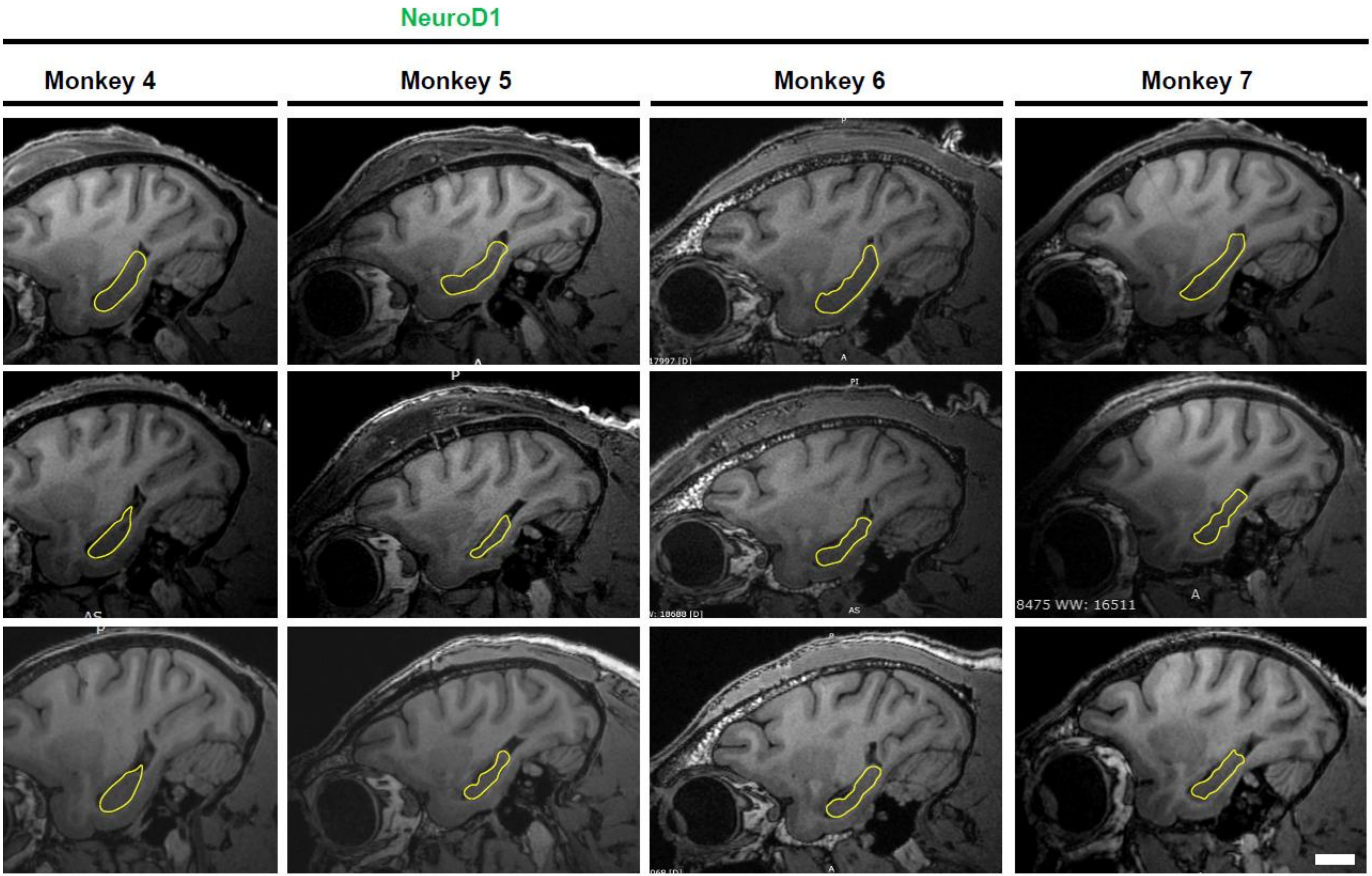
NeuroD1
Monkey 4
Monkey 5
Monkey 6
Monkey 7
8475 WW: 16511

Figure S6. NeuroD1 AAV-based gene therapy inhibits hippocampal atrophy in AD-like monkeys.

Representative sagittal sections from T1-weighted MRI scans reveal progressive hippocampal atrophy in NHP AD models from the control group, whereas hippocampal atrophy was halted in NHP AD models from the NeuroD1 treatment group. The yellow lines outline the boundaries of the hippocampus. Scale bar, 1 cm.

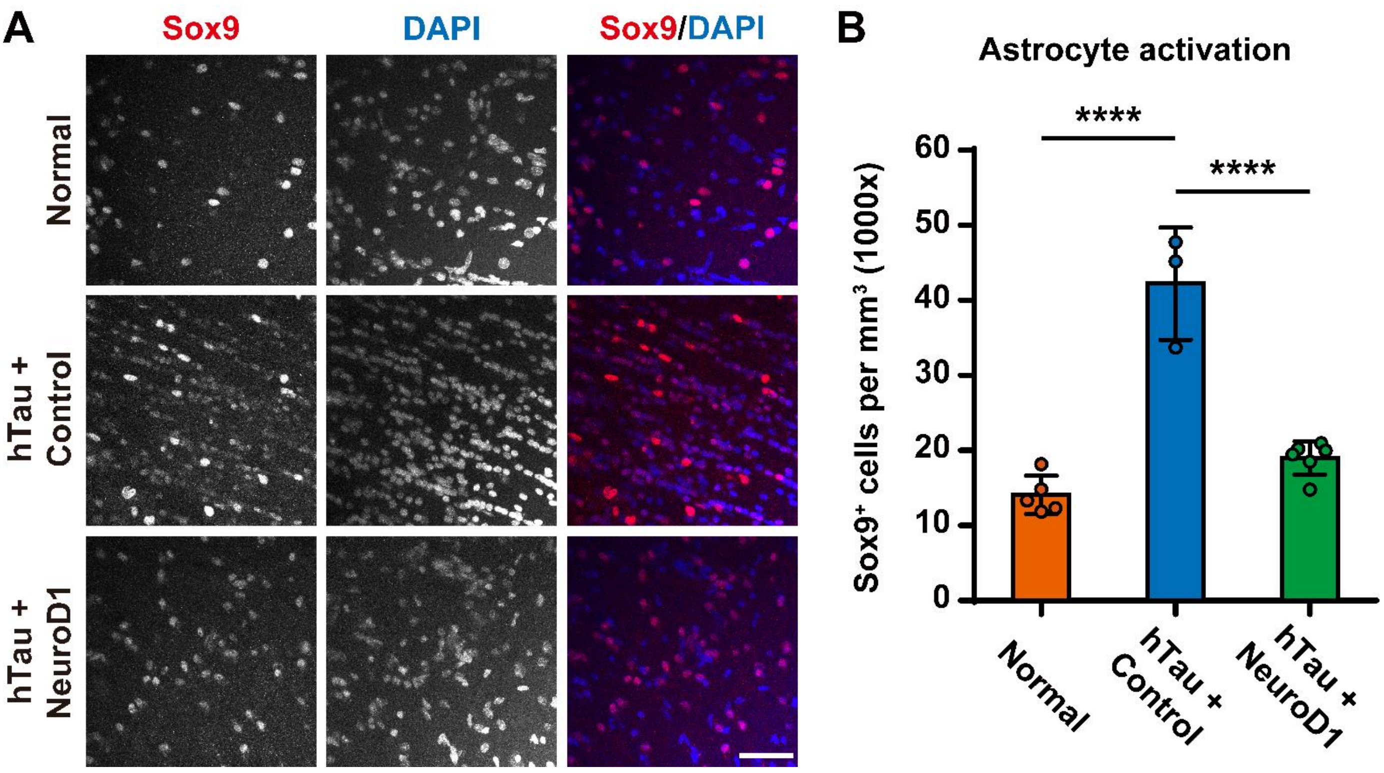

Figure S7. NeuroD1 AAV-based gene therapy reduces reactive astrogliosis in AD-like monkeys.

A) Representative images of Sox9 immunostaining demonstrate astrocyte activation following hTau overexpression and a significant decrease in astrocyte activation subsequent to NeuroD1 overexpression. Scale bar, 50 μm.

B) Quantification of the density of Sox9^{+} cells in the hippocampus of NHP AD models. ****$P < 0.0001$, One-way ANOVA with Tukey's post hoc test, Normal group $N = 5$, Control group $N = 3$, NeuroD1 group $N = 6$.

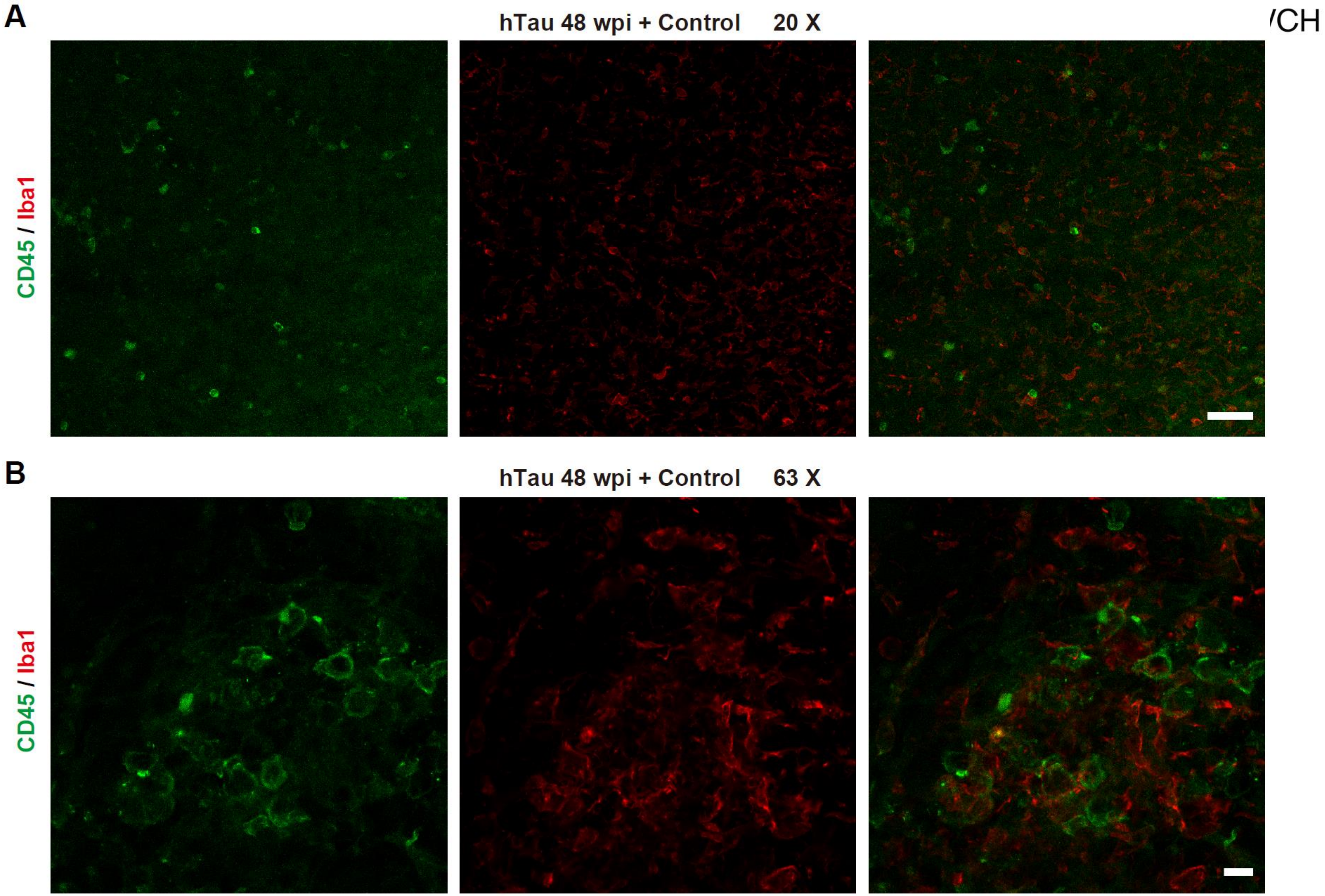

A
hTau 48 wpi + Control 20 X
B
hTau 48 wpi + Control 63 X
CD45 / Iba1
CD45 / Iba1

Figure S8. The CD45 positive cells are unlikely to be microglia in the brain slices of our AD-like monkeys.

A) Representative images of CD45 immunostaining (20X) suggest that leukocyte infiltration and macrophage proliferation following hTau overexpression in monkey hippocampus. Note that most $CD45^+$ cells are Iba1 negative. Scale bar, 50 μm.

B) Representative images of CD45 immunostaining (63X) indicate that leukocyte infiltration and macrophage proliferation following hTau overexpression in monkey hippocampus. Note that most $CD45^+$ cells are Iba1 negative. Scale bar, 10 μm.

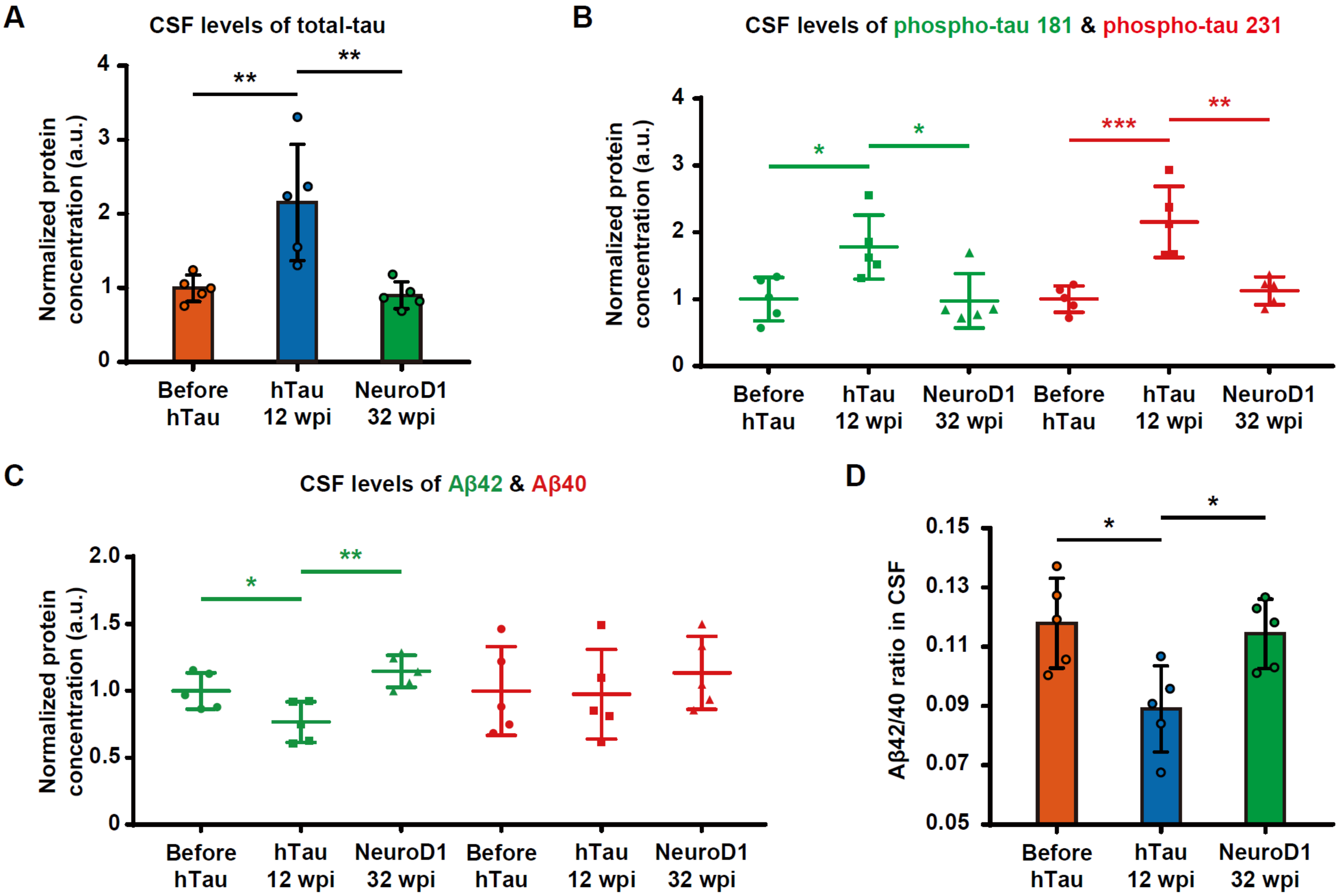
A
CSF levels of total-tau
Normalized protein concentration (a.u.)
**
**
0
1
2
3
4
Before hTau
hTau 12 wpi
NeuroD1 32 wpi
B
CSF levels of phospho-tau 181 & phospho-tau 231
Normalized protein concentration (a.u.)
*
*
***
**
0
1
2
3
4
Before hTau
hTau 12 wpi
NeuroD1 32 wpi
Before hTau
hTau 12 wpi
NeuroD1 32 wpi
C
CSF levels of Aβ42 & Aβ40
Normalized protein concentration (a.u.)
*
**
0
0.5
1.0
1.5
2.0
Before hTau
hTau 12 wpi
NeuroD1 32 wpi
Before hTau
hTau 12 wpi
NeuroD1 32 wpi
D
Aβ42/40 ratio in CSF
*
*
0.05
0.07
0.09
0.11
0.13
0.15
Before hTau
hTau 12 wpi
NeuroD1 32 wpi

Figure S9. NeuroD1 AAV-based gene therapy partially restores the levels of CSF AD biomarkers in AD-like monkeys.

A) Quantification of normalized CSF levels of total-tau detected using Simoa before and after 12 weeks of hTau expression, as well as after 32 weeks of NeuroD1 expression. $^{**}P < 0.01$, One-way ANOVA with Tukey's post hoc test, $N = 5$.

B) Quantification of normalized CSF levels of phospho-tau 181 and phospho-tau 231 detected using Simoa before and after 12 weeks of hTau expression, as well as after 32 weeks of NeuroD1 expression. $^{***}P < 0.001$, $^{**}P < 0.01$, $^{*}P < 0.05$, One-way ANOVA with Tukey's post hoc test, $N = 5$.

C) Quantification of normalized CSF levels of Aβ42 and Aβ40 detected using Simoa before and after 12 weeks of hTau expression, as well as after 32 weeks of NeuroD1 expression. $^{**}P < 0.01$, $^{*}P < 0.05$, One-way ANOVA with Tukey's post hoc test, $N = 5$.

D) Quantification of the Aβ42/Aβ40 ratio in CSF detected using Simoa before and after 12 weeks of hTau expression, as well as after 32 weeks of NeuroD1 expression. $^{*}P < 0.05$, One-way ANOVA with Tukey's post hoc test, $N = 5$.

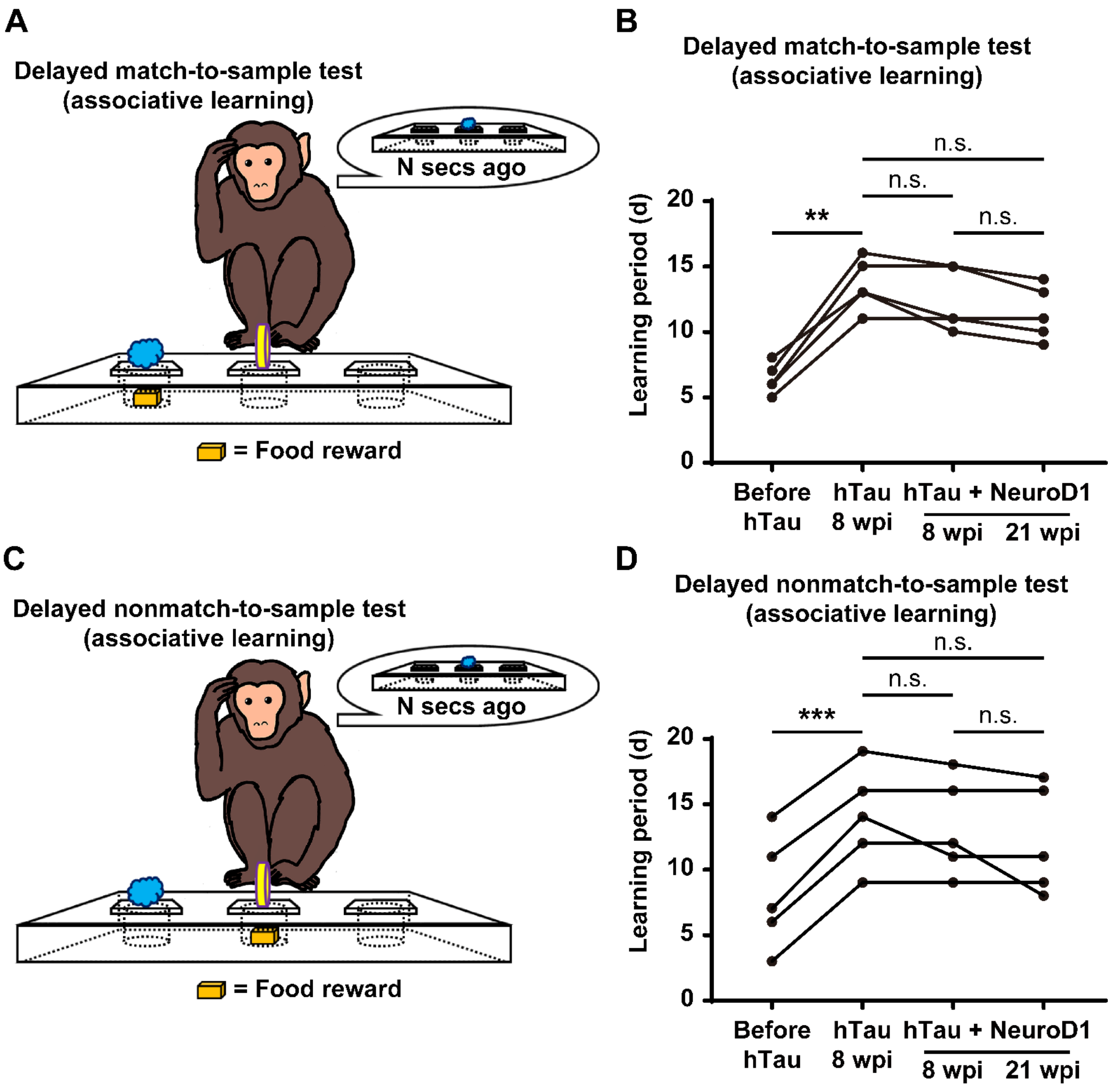

Figure S10. The delayed match-to-sample and nonmatch-to-sample tests on AD-like monkeys treated with NeuroD1 AAV-based gene therapy.

A&C) Schematic diagrams depict the "delayed match-to-sample" and "delayed nonmatch-to-sample" tasks, utilizing the WGTA to assess the "learning period" and thereby evaluate the associative learning ability of the NHPs before and after hTau overexpression, as well as following NeuroD1 AAV-based gene therapy.

B&D) Quantitation of the “ learning period” recorded in the monkeys performing the “delayed match-to-sample” task (B) and “delayed nonmatch-to-sample” task (D). Each polyline represents a distinct animal. $^{**}P < 0.01$, $^{***}P < 0.001$, “n.s.” stands for “not statistically significant”, Repeated measures ANOVA with Tukey’s post hoc test, N = 5.

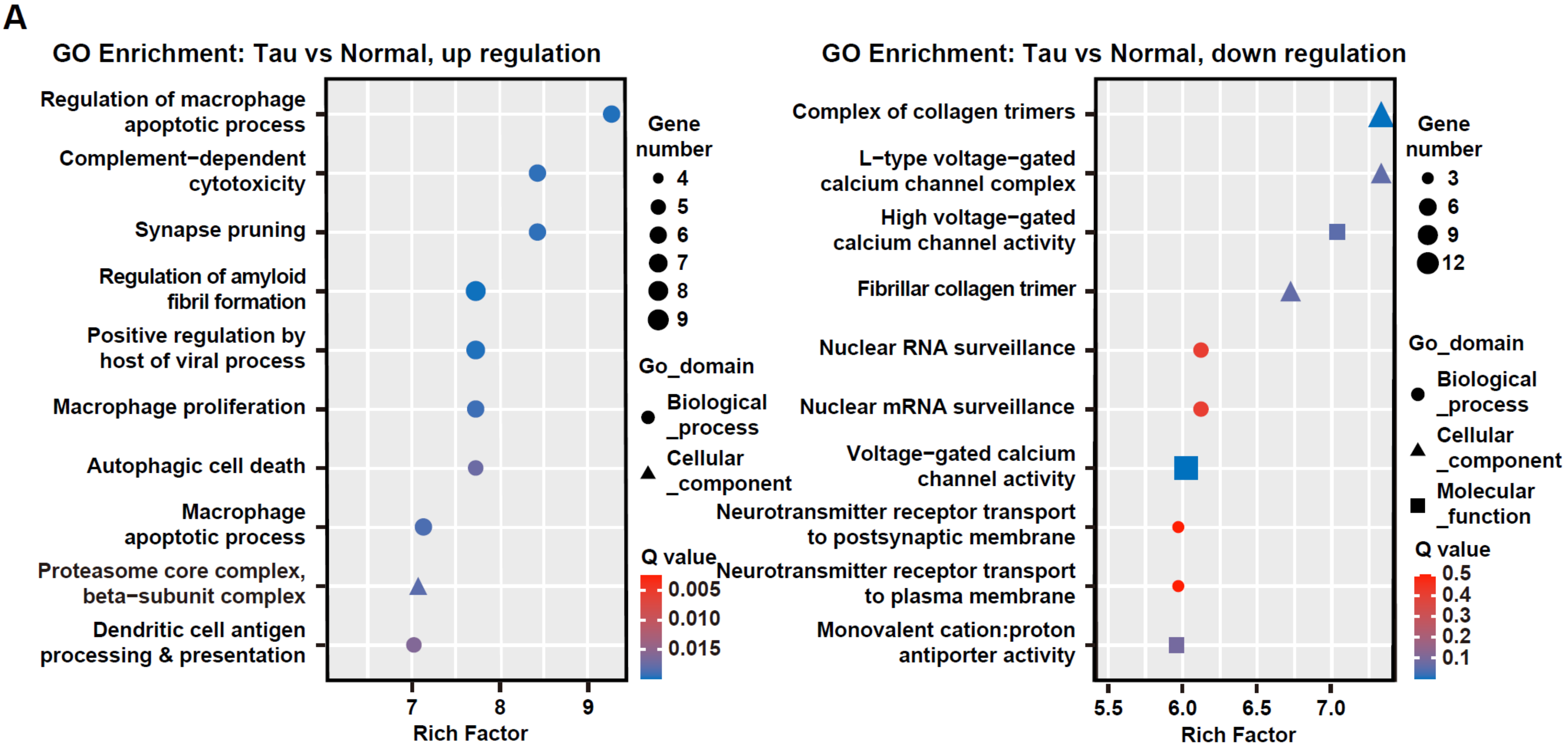
A
GO Enrichment: Tau vs Normal, up regulation
Regulation of macrophage apoptotic process
Complement−dependent cytotoxicity
Synapse pruning
Regulation of amyloid fibril formation
Positive regulation by host of viral process
Macrophage proliferation
Autophagic cell death
Macrophage apoptotic process
Proteasome core complex, beta−subunit complex
Dendritic cell antigen processing & presentation
7
8
9
Rich Factor
Gene number
4
5
6
7
8
9
Go_domain
Biological _process
Cellular _component
Q value
0.005
0.010
0.015
GO Enrichment: Tau vs Normal, down regulation
Complex of collagen trimers
L−type voltage−gated calcium channel complex
High voltage−gated calcium channel activity
Fibrillar collagen trimer
Nuclear RNA surveillance
Nuclear mRNA surveillance
Voltage−gated calcium channel activity
Neurotransmitter receptor transport to postsynaptic membrane
Neurotransmitter receptor transport to plasma membrane
Monovalent cation:proton antiporter activity
5.5
6.0
6.5
7.0
Rich Factor
Gene number
3
6
9
12
Go_domain
Biological _process
Cellular _component
Molecular _function
Q value
0.5
0.4
0.3
0.2
0.1

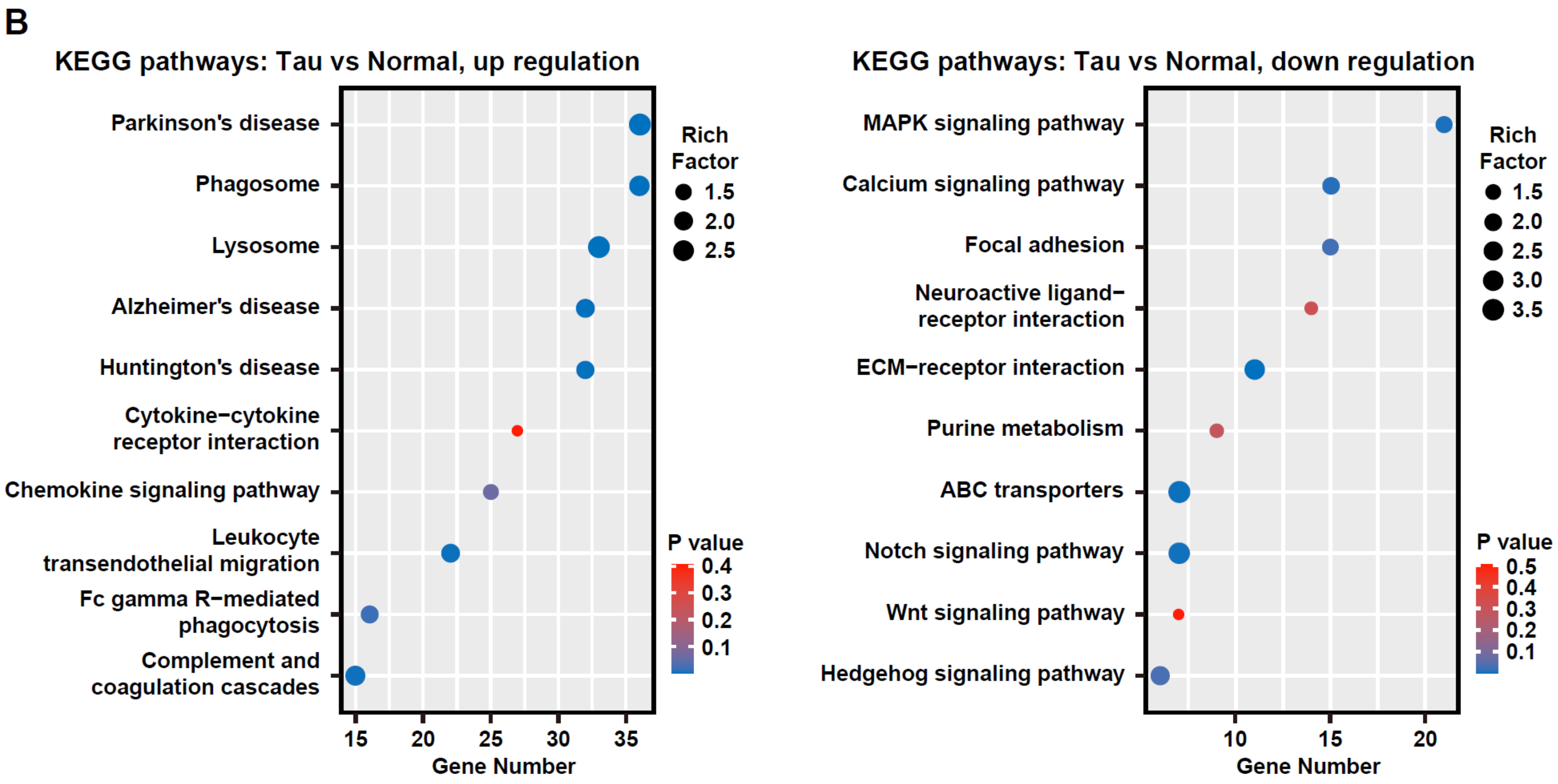
B
KEGG pathways: Tau vs Normal, up regulation
Parkinson's disease
Phagosome
Lysosome
Alzheimer's disease
Huntington's disease
Cytokine−cytokine receptor interaction
Chemokine signaling pathway
Leukocyte transendothelial migration
Fc gamma R−mediated phagocytosis
Complement and coagulation cascades
15
20
25
30
35
Gene Number
Rich Factor
1.5
2.0
2.5
P value
0.4
0.3
0.2
0.1
KEGG pathways: Tau vs Normal, down regulation
MAPK signaling pathway
Calcium signaling pathway
Focal adhesion
Neuroactive ligand−receptor interaction
ECM−receptor interaction
Purine metabolism
ABC transporters
Notch signaling pathway
Wnt signaling pathway
Hedgehog signaling pathway
10
15
20
Gene Number
Rich Factor
1.5
2.0
2.5
3.0
3.5
P value
0.5
0.4
0.3
0.2
0.1

Figure S11. Upregulation of neuroinflammation & apoptosis and downregulation of neurodevelopment & synaptic transmission in AD-like monkeys revealed by transcriptome analysis.

A) Bubble plots of GO enrichment analysis of DEGs. Top 10 GO terms are enriched. Rich factor demonstrates the degree of enrichment by GO. The node size represents the number of selected genes, and color represents the Q value of the enrichment analysis. Q value is a multiple hypothesis-corrected p value.

B) Bubble plots of KEGG pathway analysis of DEGs. Top 10 KEGG pathways are enriched. The node size represents Rich factor, and color represents the p value of the enrichment analysis. Rich factor is the ratio of the number of DEGs annotated in a pathway (as indicated in the y-axis) to the number of all genes annotated in this pathway.

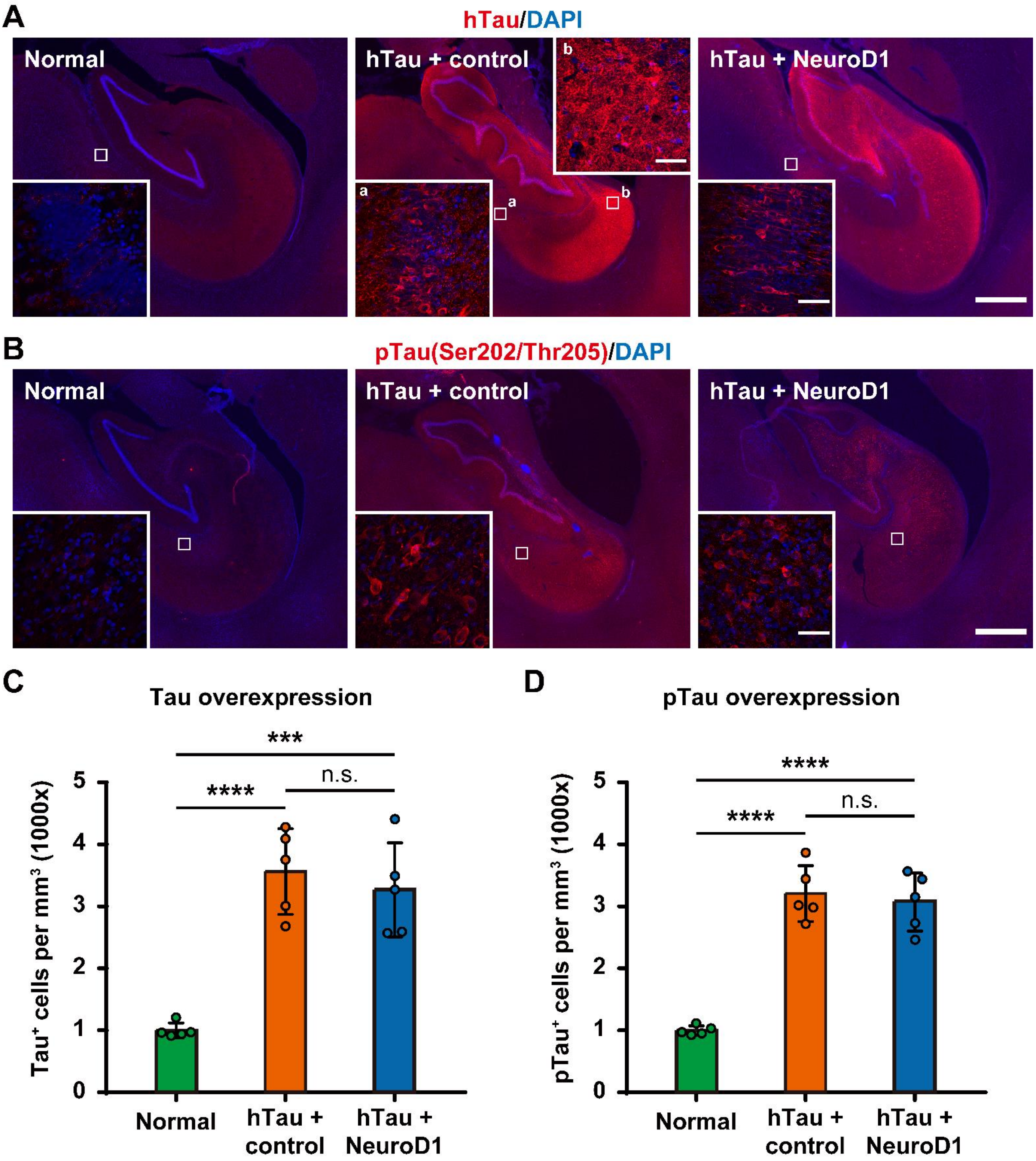

Figure S12. Tau pathology in the hippocampus of normal monkeys, AD-like monkeys, and AD-like monkeys treated with NeuroD1 AAV-based gene therapy.

A) Representative images of tau immunostaining demonstrate the AAV-induced overexpression of tau in neurons within the monkey hippocampus with or without NeuroD1 overexpression. Note the substantial

neuronal loss observed in the area with high tau expression (b). Insets show higher magnification images. Scale bar, 1 mm and 50 µm (insets).

B) Representative images of phospho-Tau (Ser202/Thr205) immunostaining exhibit excessive tau phosphorylation in neurons within the monkey hippocampus with or without NeuroD1 overexpression. Insets show higher magnification images. Scale bar, 1 mm and 50 µm (insets).

C) Quantification of the density of Tau^+ cells in monkey hippocampus. ***$P < 0.001$, ****$P < 0.0001$, "n.s." stands for "not statistically significant", One-way ANOVA with Tukey's post hoc test, $N = 5$.

D) Quantification of the density of $pTau^+$ cells in monkey hippocampus. ****$P < 0.0001$, "n.s." stands for "not statistically significant", One-way ANOVA with Tukey's post hoc test, $N = 5$.

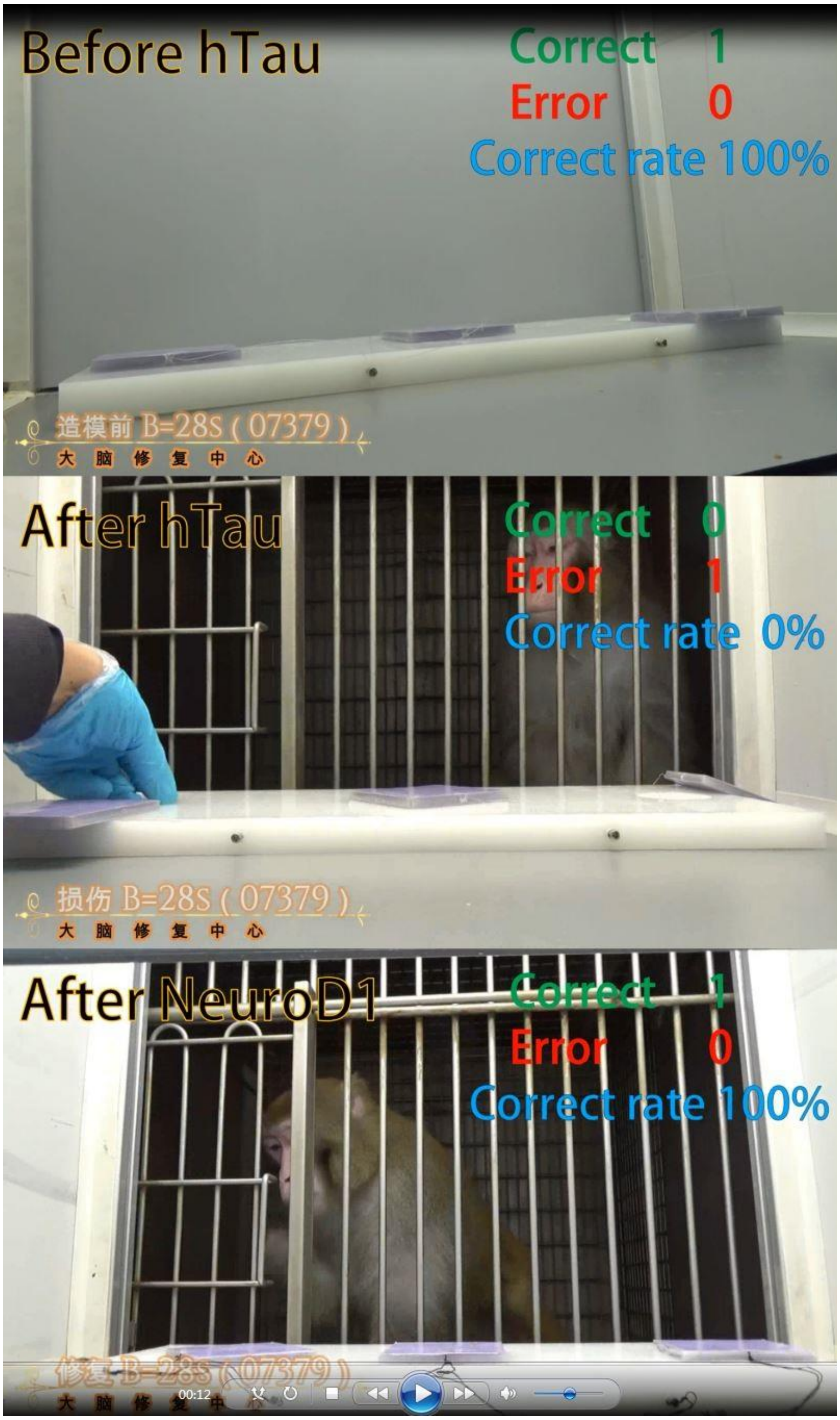

Supplementary Movie Still

Supplementary Movie. WGTA-based spatial working memory task for monkeys.

Monkey sat in front of a tray with 3 covered food wells. Experimenter baited one well, covered it, and blocked tray with screen. After designated delay, screen was removed, and monkey retrieved food from baited well using working memory. Food was randomly placed in wells over 30 trials per day, with delay durations gradually increasing as monkey mastered the task.
